# Systematic investigation of the material basis, effectiveness and safety of *Thesium chinense* Turcz. and its preparation Bairui Granules against lung inflammation

**DOI:** 10.1186/s13020-024-00940-y

**Published:** 2024-05-08

**Authors:** Guang-Cheng Peng, Jin-Hua Hao, Yue-Qin Guan, Ying-Yue Wang, Ming-Jie Liu, Guo-Hui Li, Zhen-Peng Xu, Xue-Sen Wen, Tao Shen

**Affiliations:** 1 Key Lab of Chemical Biology (MOE), School of Pharmaceutical Sciences, Cheeloo College of Medicine, Shandong University, 44 Wenhua Xi Road, Jinan, 250012 People’s Republic of China; 2 Jiuhua Huayuan Pharmaceutical Co., Ltd., Chuzhou, People’s Republic of China; 3 Department of Pharmacy, Jinan Maternity and Child Care Hospital Affiliated to Shandong First Medical University, Jinan, People’s Republic of China

**Keywords:** *Thesium chinense* Turcz., Flavonoid, Bioactive constituents, Chemical composition, Inflammation

## Abstract

**Background:**

*Thesium chinense* Turcz. (Named as Bai Rui Cao in Chinese) and its preparations (e.g., Bairui Granules) have been used to treat inflammatory diseases, such as acute mastitis, lobar pneumonia, tonsillitis, coronavirus disease 2019 (COVID-19), and upper respiratory tract infection. However, the material basis, pharmacological efficiency, and safety have not been illustrated.

**Methods:**

Anti-inflammatory activity-guided isolation of constituents has been performed using multiple column chromatography, and their structures were elucidated by NMR spectroscopy and ECD calculations. The inhibitory effects on lung inflammation and safety of the crude ethanol extract (CE), Bairui Granules (BG), and the purified active constituents were evaluated using lipopolysaccharide (LPS)-stimulated acute lung inflammation (ALI) mice model or normal mice.

**Results:**

Seven new compounds (**1**–**7**) and fifty-six known compounds (**8**–**63**) were isolated from *T. chinense*, and fifty-four were reported from this plant for the first time. The new flavonoid glycosides **1**–**2**, new fatty acids **4**–**5**, new alkaloid **7** as well as the known constituents including flavonoid aglycones **8–11**, lignans **46–54**, alkaloids **34** and **45**, coumarins **57**, phenylpropionic acids **27**, and simple aromatic compounds **39**, **44** and **58** exhibited anti-inflammatory activity. Network pharmacology analysis indicated that anti-inflammation of *T. chinense* was attributed to flavonoids and alkaloids by regulating inflammation-related proteins (e.g., TNF, NF-κB, TGF-β). Furthermore, constituents of *T. chinense* including kaempferol-3-*O*-glucorhamnoside (KN, also named as Bairuisu I, **19**), astragalin (AG, Bairuisu II, **12**), and kaempferol (KF, Bairuisu III, **8**), as well as CE and BG could alleviate lung inflammation caused by LPS in mice by preventing neutrophils infiltration and the expression of the genes for pro-inflammatory cytokines NLRP3, caspase-1, IL-1β, and COX-2. After a 28-day subacute toxicity test, BG at doses of 4.875 g/kg and 9.750 g/kg (equivalent to onefold and twofold the clinically recommended dose) and CE at a dose of 11.138 g/kg (equivalent to fourfold the clinical dose of BG) were found to be safe and non-toxic.

**Conclusions:**

The discovery of sixty-three constituents comprehensively illustrated the material basis of *T. chinense*. *T. chinense* and Bairui Granules could alleviate lung inflammation by regulating inflammation-related proteins and no toxicity was observed under the twofold of clinically used doses.

**Supplementary Information:**

The online version contains supplementary material available at 10.1186/s13020-024-00940-y.

## Introduction

Acute lung inflammation (ALI) or acute respiratory distress syndrome (ARDS) has a high global incidence rate and mortality [1, 2]. The increased permeability of alveolar capillary barrier, which causes pulmonary edema and impaired arterial oxygenation, are symptoms of ALI/ARDS [3]. ALI/ARDS can be caused by a variety of conditions, such as bacterial or viral infection, acute eosinophilic pneumonia and immunologically mediated pulmonary hemorrhage [4]. During the global period of coronavirus disease 2019 (COVID-19), a survey showed that 29% of patients with infectious COVID-19 developed into ARDS [5].

*Thesium chinense* Turcz., known as “Bai Rui Cao”(百蕊草) in Chinese, is a kind of perennial weak herb distributed in northeast China, North China, and most areas south of the Yangtze River. It is a parasitic plant that feeds on the roots of other plants, and is born on the edge of grassland and sand dunes between 500 and 2700 m above sea level. This folk herbal medicine is originally recorded in the ancient Chinese medicine book “*Bencao Tujing*” (本草图经) [6, 7]. As a traditional Chinese medicine (TCM), the whole herb is mainly used to treat various inflammatory diseases, such as acute mastitis, lobar pneumonia, tonsillitis, and upper respiratory tract infection [6, 8].

Phytochemical and pharmacological aspects of *T. chinense* has not been extensively studied. Hitherto, only about 60 compounds including flavonoids, alkaloids, phenylpropanoids, steroids, and fatty acids, have been identified, and flavonoids are recognized as the main chemical constituents of *T. chinense* [9–15]. Pharmacological test indicated that *T. chinense* inhibited mouse auricle swelling caused by xylene, rat foot swelling caused by egg white, rat cotton ball pulp odontoma, and mice’s ear edema caused by xylene [16, 17].

Contemporary pharmaceutics preparation of *T. chinense* has been utilized to treat pulmonary inflammation-related diseases. Administration of Bairui Granules alone or combined with other drugs (e.g., amoxicillin) could shorten the course of acute tonsillitis [18], and significantly improve symptoms in children with acute bronchitis [19]. Bairui Granules as adjunctive therapy effectively alleviate clinical symptoms of severe childhood pneumonia, promoting faster recovery in pediatric patients [20]. Bairui Granules could alleviate the inflammatory response of chronic obstructive pulmonary disease (COPD) patients and improve the clinical efficacy [21]. Since its remarkable anti-inflammatory and anti-infective effects, Bairui Granules were recommended by two official TCM treatment guidelines to treat acute pharyngitis, and laryngitis [22, 23]. Noteworthingly, during the COVID-19 pandemic, Bairui Granules was included in the official drug catalog issued by local governments of China, including Beijing, Shandong, Hebei, Anhui, etc., and was suggested to be the preferred medicine for the treatment of COVID-19 infection.

Although it has a good therapeutic effect against respiratory diseases in the TCM, the research on chemical constituents and their anti-inflammatory effect are still insufficient. In this work, anti-inflammatory constituents were identified using the bioactivity-guided strategy, and the anti-inflammatory effects of CE, BG and purified constituents were evaluated using the LPS-induced ALI model in vivo. Collectively, we found a series of active compounds from *T. chinense* that support its traditional application as an anti-inflammatory agent. The safety of *T. chinense* and Bairui Granules has been investigated in vivo.

## Materials and methods

### General

^1^H NMR, ^13^C NMR and 2D NMR spectra were acquired on a Bruker Avance DRX-600 spectrometer (600 MHz for ^1^H NMR and 150 MHz for ^13^C NMR). HRESIMS spectra were obtained from an LTQ Orbitrap XL mass spectrometer (Thermo Fisher scientific). The optical rotations were measured by GYROMAT-HP polarimeter (Anton paar). The ECD spectra were carried out on an applied Photophysics Chirascan spectrometer. Semipreparative HPLC was performed on an Agilent 1260 instrument equipped with a diode array detector (DAD) and a YMC-Pack ODS-A column. For column chromatography, silica gel (200–300 mesh, Qingdao Haiyang Chemical Group Corporation), Sephadex LH-20 (25–100 µm, Pharmacia Bio-teck), MCI gel (CHP20/P120, 120 µm, Mitsubishi chemical corporation), and RP-C_18_ silica gel (75 µm, YMC ODS-A) were used. TLC was carried out with silica gel GF_254_ plates (Qingdao Haiyang Chemical Group Corporation).

### Chemicals and reagents

LPS was obtained from Sigma-Aldrich. 3,4-Dihydroxy-benzohydroxamic acid (DIDOX) was obtained from MedChem Express. 3-(4,5-dimethyl-2-thiazolyl)-2,5-Diphenyl-2-H-tetrazolium bromide (MTT), and glycerol were purchased from Genview. Dexamethasone (DEX) was purchased from Cisen pharmaceutical company. Fetal bovine serum (FBS) was obtained from Gemini Bio-product. Naphthylethylenediamine and sulfanilamide were obtained from Sinopharm Chemicals. L-glutamine was obtained from Solarbio. Dulbecco’s Modified Eagle’s Medium (DMEM) was obtained from Gibco. In addition to the HPLC was used for chromatography-grade solvents (Tianjin Concord Technology Co., Ltd), other solvents were analytical grade (Tianjin Fuyu Fine Chemical Co., Ltd).

### Plant material

The plant materials were collected from Fuyang, Anhui, P. R. China, in July 2019 and authenticated by Prof. Xue-Sen Wen (School of Pharmaceutical Sciences, Shandong University). Voucher specimens (20,190,909–10-BRC) were deposited in the Laboratory of Pharmacognosy, School of Pharmaceutical Sciences, Shandong University, China.

### Extraction and isolation of anti-inflammatory activity guided screening

The whole herbs of dried *T. chinense* (12.0 kg) were crushed and soaked at room temperature for 3 h. Then it was extracted by heat reflux with 75% ethanol for 3 times (each time for 3 h). After filtrating, the residue was concentrated in vacuo on a rotary evaporator to obtain a crude ethanol extract of 2850.0 g (yield 23.8%), which had no obvious nitric oxide (NO) production inhibitory activity. The extract of 1800.0 g was suspended in H_2_O and partitioned with petroleum ether (PE), dichloromethane (DCM) and ethyl acetate (EtOAc) for 4 times, respectively. Finally, 41.2 g of PE fraction, 186.5 g of DCM fraction, 35.1 g of EtOAc fraction and 1537.2 g of H_2_O fraction were obtained. The EtOAc fraction was selected for the further trace of activity, because of the inhibitory activity of the fraction on LPS-induced NO production in RAW 264.7 cells (Additional file 1: Fig. S1). The EtOAc fraction (35.0 g) was subjected to silica gel column eluted with PE − EtOAc (1:0 → 0:1) and EtOAc − MeOH (1:0 → 0:1) to obtain eight fractions (E1-E8). Among these fractions, E3-E7 displayed significant inhibitory effect against NO production and thus were selected for the further trace (Additional file 1: Fig. S1). The detailed isolation process was shown in the Supplementary Data.

Compounds **4**, **5**, **8**, **26–28**, **27**, **37**, **38**, **39**–**42** and **43** were isolated from E3. Compounds **9**–**11**, **25**, **32**, **35**, **36** and **43** were obtained from E4. Compounds **16**–**18** and **30** were afforded from E5. Compounds **12**, **14**, **15**, **22**, **24** and **38** were obtained from E6. E7 was separated to yield **1–3**, **13**, **19–21**, **23**, **29**, **31**, **33** and **34**.

The other whole herbs of dried *T. chinense* (5.0 kg) were crushed and then extracted by 0.3 mol/L HCl. The acidic solution was adjusted to pH 10 with 1.32 mol/L NaOH and extracted with DCM to obtain the water extract (11.3 g, yield 0.23%). The water extract had better anti-inflammatory activity (Additional file 1: Fig. S1) than the ethanol extract and 5.2 g water extract was subjected to a silica gel column eluted with PE − DCM − NH_3_·H_2_O (1:1:0.03) and DCM − MeOH (97: 3 → 6:4) to give 10 fractions (S1–S10). The results indicated that the fractions of S5, S6 and S7 had significant NO production inhibitory activities (Additional file 1: Fig. S1). Compounds **6**,** 7**, **45**, **58** and **59** were obtained from S5. S6 was isolated to yield **48**, **49**, **60**, **62** and **63**. Compounds **46**, **47**, **50–57** and **61**, were obtained from S7.

### Physical and spectroscopic data of new compounds

#### Compound 1

Thesiuside A. Yellow oil; [α]_D_^20^ + 60.3 (c 1.0, MeOH); ^1^H NMR (CD_3_OD, 600 MHz) and ^13^C NMR (CD_3_OD, 150 MHz) data see Table 1; HRESIMS *m/z* 879.1991 [M + H] ^+^ (calcd. for 879.1978).

**Table 1:** ^1^H NMR and ^13^C NMR spectroscopic data for compounds** 1**–**2**

No	**1**		**2**	
position	***δ***_**C**_	***δ***_**H**_	***δ***_**C**_	***δ***_**H**_
2	119.5		119.5	
3	81.7		81.7	
4	192.5		192.5	
5	165.5		165.6	
6	98.0	5.96, d (2.1)	98.0	5.92, d (2.1)
7	169.6		169.6	
8	96.2	5.90, d (2.1)	96.2	5.88, d (2.1)
9	162.6		162.6	
10	100.3		100.2	
1′	125.2		125.1	
2′/6′	129.6	7.35, d (8.9)	129.6	7.36 d (8.9)
3′/5′	115.8	6.78, d (8.9)	115.8	6.78 d (8.9)
4′	160.2		160.2	
2′′	158.8		158.6	
3′′	134.7		134.7	
4′′	179.6		179.7	
5′′	166.6		166.6	
6′′	96.3	6.57, s	96.2	6.57, s
7′′	167.1		167.1	
8′′	108.1		108.1	
9′′	153.5		153.4	
10′′	107.4^b^		107.3^b^	
1′′′	122.5		122.5	
2′′′/6′′′	132.9	8.12, d (9.0)	132.9	8.20, d (9.0)
3′′′/5′′′	116.0	6.90, d (9.0)	115.9	6.91, d (9.0)
4′′′	161.7		161.7	
1′′′′(Glc)	100.1	5.74, d (7.7)	100.1	5.80, d (7.7)
2′′′′	79.7	3.63, dd (9.2, 7.7)	80.27	3.63, dd (9.2, 7.7)
3′′′′	79.0	3.55, dd (9.2, 8.7)	78.9	3.53, dd (9.2, 8.7)
4′′′′	71.9	3.27, dd (9.8, 8.7)	71.8	3.24, dd (9.8, 8.7)
5′′′′	78.5	3.23, ddd (9.8, 5.9, 2.1)	78.6	3.19, ddd (9.8, 5.9, 2.1)
6′′′′a	62.7	3.73, dd (12.0, 2.1)	62.5	3.70, dd (12.1, 2.1)
6′′′′b		3.49, dd (12.0, 5.9)		3.43, dd (12.1, 5.9)
1′′′′′(Rha)	102.5	5.23, d (1.9)	102.8	5.22, d (1.9)
2′′′′′	72.4	3.98, dd (3.4, 1.9)	72.4	4.00, dd (3.4, 1.9)
3′′′′′	72.3	3.73, dd (9.5, 3.4)	72.3	3.76, dd (9.5, 3.4)
4′′′′′	74.0	3.33, overlap	74.0	3.33, overlap
5′′′′′	69.9	3.98, overlap	69.9	4.01, overlap
6′′′′′	17.5	0.91, d (6.2)	17.5	0.93, d (6.2)

#### Compound 2

Thesiuside B. Yellow oil; [α]_D_^20^ -157.2 (c 1.0, MeOH); ^1^H NMR (CD_3_OD, 600 MHz) and ^13^C NMR (CD_3_OD, 150 MHz) data see Table 1; HRESIMS *m/z* 879.1991 [M + H] ^+^ (calcd. for 879.1978).

#### Compound 3

4-acetyl-6, 4′-diferuloylsucrose. Colorless amorphous solid; [α]_D_^20^ + 103.3 (c 1.0, MeOH); ^1^H NMR (CD_3_OD, 600 MHz) and ^13^C NMR (CD_3_OD, 150 MHz) data see Table 2; HRESIMS *m/z* 735.2159 [M − H] ^−^ (calcd. for 735.2141).

**Table 2:** ^1^H NMR and ^13^C NMR spectroscopic data for compound **3**

No. position		**3**	No. position	**3**	
	***δ***_**C**_	***δ***_**H**_		***δ***_**C**_	***δ***_**H**_
1	63.6	3.72, s	1′′	127.7	
2	105.9		2′′	112.0	7.13, d (2.0)
3	78.9	4.19, d (8.2)	3′′	149.3	
4	76.5	4.14 overlap	4′′	150.7	
5	81.0	4.03 overlap	5′′	116.5	6.80, d (8.2)
6	66.4	4.48, d (6.8) 4.47, d (3.7)	6′′	124.2	7.03, dd (8.2, 2.0)
OAc-4	172.7 20.8	2.04, s	α′′′	147.4	7.63, d (16.0)
1′	93.5	5.47, d (3.8)	β′′′	115.2	6.33, d (16.0)
2′	73.4	3.58, dd (9.7, 3.8)	γ′′′	168.3	
3′	72.6	4.02, dd (9.7, 9.2)	1′′′	127.6	
4′	72.6	4.93, dd (10.2, 9.2)	2′′′	111.8	7.12, d (2.0)
5′	69.9	4.40, ddd (10.2, 5.5, 2.9)	3′′′	149.3	
6′	64.5	4.15 dd (12.1, 5.5) 4.13 dd (12.1, 2.9)	4′′′	150.7	
α′′	147.1	7.62, d (16.0)	5′′′	116.5	6.78, d (8.2)
β′′	115.2	6.39, d (16.0)	6′′′	124.1	7.02, dd (8.2, 2.0)
γ′′	169.0		OMe	56.5	3.87, s
OMe	56.5	3.86, s			

#### Compound 4

(2*E*, 4*E*, 6*R*)-6-hydroxy-2,4-oxtadienoic acid. Yellow oil; [α]_D_^20^ -3.3 (c 1.0, MeOH); ^1^H NMR (CD_3_OD, 600 MHz) and ^13^C NMR (CD_3_OD, 150 MHz) data see Table 3; HRESIMS *m/z* 155.0706 [M − H] ^−^ (calcd. for 155.0714).

**Table 3:** ^1^H NMR and ^13^C NMR spectroscopic data for compounds** 4–5**

No	**4**		**5**	
position	***δ***_**C**_	***δ***_**H**_	***δ***_**C**_	***δ***_**H**_
1	171.8		169.7	
2	124.0	5.89, d (15.4)	131.5	6.10, d (15.9)
3	144.8	7.22, dd (15.4, 11.0)	126.8	6.69, dt (15.9, 2.0)
4	128.8	6.39, dd (15.2, 11.0)	78.8	
5	145.8	6.10, dd (15.2, 6.1)	101.2	
6	74.0	4.08, q (6.1)	20.2	2.40, td (7.0, 2.0)
7	30.9	1.56, m	29.4	1.56, m
8	10.1	0.94, t (7.4)	29.7	1.35, m
9			29.8	1.35, m
10			30.0	1.42, m
11			25.9	1.62, m
12			34.7	2.32. t (7.4)
13			176.0	
OMe			52.0	3.65, s

#### Compound 5

(*E*)-13-methoxy-13-oxotridec-2-en-4-ynoic acid. Colorless amorphous solid; [α]_D_^20^ -1.8 (c 1.0, MeOH); ^1^H NMR (CD_3_OD, 600 MHz) and ^13^C NMR (CD_3_OD, 150 MHz) data see Table 3; HRESIMS *m/z* 251.1279 [M − H] ^−^ (calcd. for 251.1289).

#### Compound 6

Thesiumine A. Colorless oil; [α]_D_^20^ -2.7 (c 1.0, MeOH); ^1^H NMR (CD_3_OD, 600 MHz) and ^13^C NMR (CD_3_OD, 150 MHz) data see Table 4; HRESIMS *m/z* 247.1805 [M + H] ^+^ (calcd. for 247.1805).

**Table 4:** ^1^H NMR and ^13^C NMR spectroscopic data for compounds **6**–**7**

No	**6**		**7**	
position	***δ***_**C**_	***δ***_**H**_	***δ***_**C**_	***δ***_**H**_
2a 2b	57.0	3.46, overlap 2.89, overlap	56.9	3.47, overlap 2.88, overlap
3a 3b	19.9	1.97, overlap 1.70, overlap	19.9	2.01, overlap 1.72, overlap
4a 4b	26.8	1.85, m 1.80, overlap	26.8	1.86, overlap 1.78, overlap
5	34.8	2.11, overlap	35.5	1.99, overlap
6	64.6	3.39, t (3.2)	64.9	3.36, t (2.9)
7	41.2	2.07, overlap	43.0	1.84, overlap
8a 8b	25.2	2.01, overlap 1.72, overlap	25.2	2.06, overlap 1.70, overlap
9a 9b	20.3	2.07, overlap 1.74, overlap	20.3	2.06, overlap 1.70, overlap
10a 10b	56.9	3.46, overlap 2.89, overlap	56.9	3.47, overlap 2.88, overlap
11	52.0	4.13, ddd, (11.8, 8.4, 7.3)	53.7	4.01, ddd (11.3, 8.9, 6.0)
12a 12b	28.0	2.79, m 2.34, m	28.1	2.22, m 1.50, m
13a 13b	140.7	6.64, ddd (9.8, 4.7, 3.8)	19.3	1.84, overlap 1.72, overlap
14a 14b	124.1	5.84, dt (9.8, 2.0)	33.4	2.39, m 2.28, m
15	167.6		172.3	
17a 17b	42.0	4.25, dd (14.0, 5.0) 3.23, dd (14.0, 12.9)	41.5	4.49, dd (14.0, 4.7) 3.18, t (14.0, 12.8)

#### Compound 7

Thesiumine B. Colorless oil; [α]_D_^20^ + 11.2 (c 1.0, MeOH); ^1^H NMR (CD_3_OD, 600 MHz) and ^13^C NMR (CD_3_OD, 150 MHz) data see Table 4; HRESIMS *m/z* 249.1960 [M + H] ^+^ (calcd. for 249.1961).

### ECD calculations

The theoretical calculations were carried out by using the Gaussian 09 program. The conformers were optimized at the B3LYP/6-31G (d, p) level according to the Boltzmann distribution theory. ECD calculations were performed using the TD-DFT method at the B3LYP/6-31G (d, p) level (in methanol solution with SMD implicit solvation model).

### Glycohydrolysis and sugar identification experiments

Compound **3** (2 mg) was hydrolyzed with 3% HCl (2 mL) and heated under reflux for 2 h. The products were subjected to identified by comparing the TLC behavior with D-glucose and D-fructose [developed with CHCl_3_-MeOH-H_2_O (9:11:2) plus two drops of glacial acetic acid]. Then, the reaction mixture was extracted by using ethyl acetate and monosaccharide residues were obtained from H_2_O layer extraction. The residues were dissolved in pyridine (0.5 mL) and heated with L-cysteine methyl ester (1.5 mg) for 2 h at 60 °C, and then aryl isothiocyanate (1.5 µL) was added and further reacted for 2 h at 60 °C. The derivatives of D-glucose and L-glucose were produced by using the same method. The reaction mixtures, derivatives of D-glucose and L-glucose were analyzed by HPLC (MeOH − H_2_O, 10:100 to 100:0).

### Anti-inflammatory activity screening in vitro

#### Cell culture

RAW 264.7 murine macrophages were purchased from the American Type Culture Collection (ATCC, USA) and cultured at 37 ℃ with 5% CO_2_ using a cell incubator. DMEM high glucose media were supplemented with 10% FBS, 1% penicillin and streptomycin for cell culture.

#### NO production inhibition assay

The RAW 264.7 cells (1 × 10^5^ cells/well) were inoculated into a 96-well plate for overnight incubation. The model group received DMEM culture with LPS (1 µg/mL), and the drug-treatment groups were exposed to DMEM medium with LPS (1 µg/mL) and indicated doses of tested drugs for 24 h. Meanwhile, the control group received DMEM medium. Subsequently, 100 µL of supernatant from each well was mixed with 100 µL of the Griess reagent in a separate 96-well plate. Absorbance at 570 nm was measured using the Model 680 plate reader (Bio-Rad, USA), with NaNO_2_ used to establish a standard curve. Then, 20 µL of MTT solution (2 mg/mL) was added to each well. After an additional 3h incubation, the supernatant was aspirated, and 100 µL of dimethyl sulfoxide (DMSO) was added to dissolve the precipitation. Cell viability was determined by measuring the absorbance at 570 nm.

### Anti-inflammatory evaluation in vivo

#### ALI model establishment and drug administration

Forty male C57BL/6N mice (6–8 weeks, 19–21 g, Beijing Vital River Laboratory Animal Technology Co., Ltd.) were utilized in this research. The mice were housed under a 12 h/12 h dark/light cycle with enough water and regular rodent diet. All in vivo experiments were conducted in accordance with the rules of Ethical Committee and Institutional Animal Care and Use Committee of Shandong University. After one-week adaptive feeding, the mice were randomly divided into eight groups (n = 5) for different treatments: the control group, the LPS group, the DEX group (1 mg/kg), the CE group (50 mg/kg), the BG group (50 mg/kg), the KN (**19**) group (20 mg/kg), the AG (**12**) group (20 mg/kg) and the KF (**8**) group (20 mg/kg). All drugs and DEX were dissolved with 5% ethanol saline. The administration of the above drugs was carried out via an intragastric administration (i.g.) 4 h after LPS intratracheal instillation for a total of 14 days. Simultaneously, a same volume of 5% ethanol saline was given to mice in the control group and LPS group. The mice were sacrificed on the 14th day.

#### Histopathological evaluation

The lung samples were collected, fixed with a 10% paraformaldehyde solution, dehydrated routinely, embedded in paraffin, sliced into sections of 4 µm thick, and stained with hematoxylin and eosin (H&E). Histopathology was evaluated under 100 × magnification using BX53 + DP73 microscope system.

#### Bronchoalveolar Lavage Fluid (BALF) analysis

After the mice were sacrificed, the trachea of the mice was fully exposed. 1 mL of pre-cooled phosphate-buffered saline (PBS) was injected into the trachea and recovered with a syringe three times to obtain the total BALF. Then, the supernatant was collected after centrifugation at 2000 rpm for 20 min under 4 °C. The level of interleukin-1β (IL-1β) in the BALF was tested by ELISA kit (ABclonal Technology Co., Ltd.) according to the manufacturer’s instructions.

#### Complete blood counts

Orbital blood of mice was gathered into EDTA anticoagulation tubes (Wuhan Servicebio Technology Co., Ltd.). Complete blood cell counts were analyzed by an automatic blood cell analyzer (mindray BC-6800).

#### Quantitative Real-Time Polymerase Chain Reaction (qRT-PCR)

The total RNA of the lung tissues was extracted by Trizol reagent (Invitrogen). Reverse transcription was performed using PrimeScriptTM RT Reagent Kits with gDNA Eraser (Takara Bio Inc., Shiga, Japan) following the instructions. qRT-PCR analysis was carried out on LightCycler 480II RT-PCR system (Roche, Basel) using primer and TB GreenTM Premix Ex TaqTM (Takara Bio Inc.). The data was relative mRNA levels normalized to β-actin.

All primer sequences are represented in Additional file 1: Table S1.

### *S*afety evaluation in vivo

#### 2.10.1. Subacute toxicity model establishment and drug administration

A total of 12 male and 12 female Kunming mice, at the age of four weeks and weighing between 18 and 22 g, were obtained from Beijing Vital River Laboratory Animal Technology Co., Ltd. They were randomly assigned to four different treatment groups (n = 6 per group): the control group (purified water), the CE group (at 11.138 g/kg), the low-dosage BG group (BG-L at 4.875 g/kg), and the high-dosage BG group (BG-H, at 9.75 g/kg). All animals were acclimatized to the laboratory conditions for one week before the study commenced.

The subacute toxicity study adhered to the guidelines outlined in the OECD guideline 407, which provides standardized procedures for evaluating the toxicity of substances. While the control group received a standard diet and purified water, the other groups were orally administered the test medicine dissolved in purified water daily for a duration of 28 days. All animals were systematically evaluated for clinical signs, including behavioral changes, and a range of physiological parameters such as body weight, urination patterns, food and water intake, respiration, convulsions, tremors, constipation and any alterations in eye and skin coloration, etc. These assessments were conducted at multiple time intervals, including 30 min, 1 h, 3 h, and 6 h after each dosing. Blood samples were collected from the mice via retro-orbital puncture under anesthesia to minimize animal discomfort. One portion of the collected blood was transferred to a centrifuge tube, while the remaining portion was placed into an anticoagulant tube containing EDTA for standard blood tests. The former portion was subjected to centrifugation at 4,000 rpm for 15 min to separate plasma, and the resulting supernatant was carefully extracted. The excised organs were rinsed with ice-cold saline solution (0.9% NaCl) to remove any residual blood, followed by drying with absorbent tissue paper, measurement of organ weights, and preservation in a 4% paraformaldehyde solution. These preserved organs were later utilized for histopathological examination. Throughout the 28-day period of oral drug administration, detailed records of mortality were meticulously maintained for each experimental group.

#### Body weight and organ coefficient

Body weight measurements were recorded every other day throughout the subacute toxicity study using a digital balance. After the 29-day study period, the animals were humanely euthanized, and all organs were carefully collected. Blood was meticulously removed from each organ, including the heart, liver, spleen, lung, kidney, stomach and colon. The weight of each organ was determined using a digital balance. Subsequently, the relative organ weight was calculated as the ratio of the organ weight to the body weight, expressed as a percentage, using the formula below:

$${\text{Organ coefficient }}\left( \% \right)\, = \,\left( {{\text{Organ Weight }}/{\text{ Body Weight}}} \right)\, \times \,100\ \%$$

#### Hematological analysis

The following hematological parameters were evaluated from the collected blood samples: red blood cell count (RBC), white blood cell count (WBC), hemoglobin concentration, hematocrit, platelet count, mean platelet volume (MPV), mean corpuscular hemoglobin (MCH), eosinophils, lymphocytes, neutrophil-to-granulocyte ratio (neut/gran). These hematological parameters were quantified using an automated blood cell analyzer (mindray BC-6800).

#### Biochemical parameters

Serum samples were analyzed for the following parameters: creatine kinase (CK), lactate dehydrogenase (LDH), total bile acids (TBA), alanine aminotransferase (ALT), aspartate aminotransferase (AST), alkaline phosphatase (ALP), blood urea nitrogen (BUN), creatinine (Crea). These biochemical parameters were quantified using an automatic biochemical analyzer (Chemray 240, Rayto Life and Analytical Sciences Co., Ltd.).

#### Histopathological assessment

Heart, liver, spleen, lung, kidney, stomach, and colon specimens were fixed in 4% paraformaldehyde. These specimens then underwent a series of processing steps, including dehydration with a graded series of alcohol concentrations (beginning with lower concentrations and ending with absolute alcohol), xylene cleaning to remove residual paraffin, paraffin embedding to prepare tissue blocks, and sealing for preservation. Subsequently, sections measuring 4 μm in thickness were cut and stained with H&E. All tissue samples were randomly examined under light microscopes at 20 × magnification using the BX53 + DP73 microscope system to comprehensively analyze and identify any histological alterations.

### Statistical analysis

The analysis was performed using Graph Pad Prism 8.0 software. One-way analysis of variance (ANOVA) and the LSD test were used for multiple comparisons. Significant level was established at P < 0.05. Data were presented as means ± SD.

## Results

### Structure elucidation of constituents

A total of sixty-three constituents, including seven new compounds (including two flavonoid glycosides **1**–**2**, one phenylpropionate **3**, two fatty acids **4**–**5** and two alkaloids **6**–**7**), were isolated and identified from *T. chinense*. The isolation and identification of new compounds have deepened our understanding of the chemical composition of *T. chinense*, enhancing the in-depth analysis of the drug-target network. This leads to a better discovery of active ingredients and a more comprehensive understanding of *T. chinense*'s mechanism of action in treating lung inflammation.

**Fig. 1:**
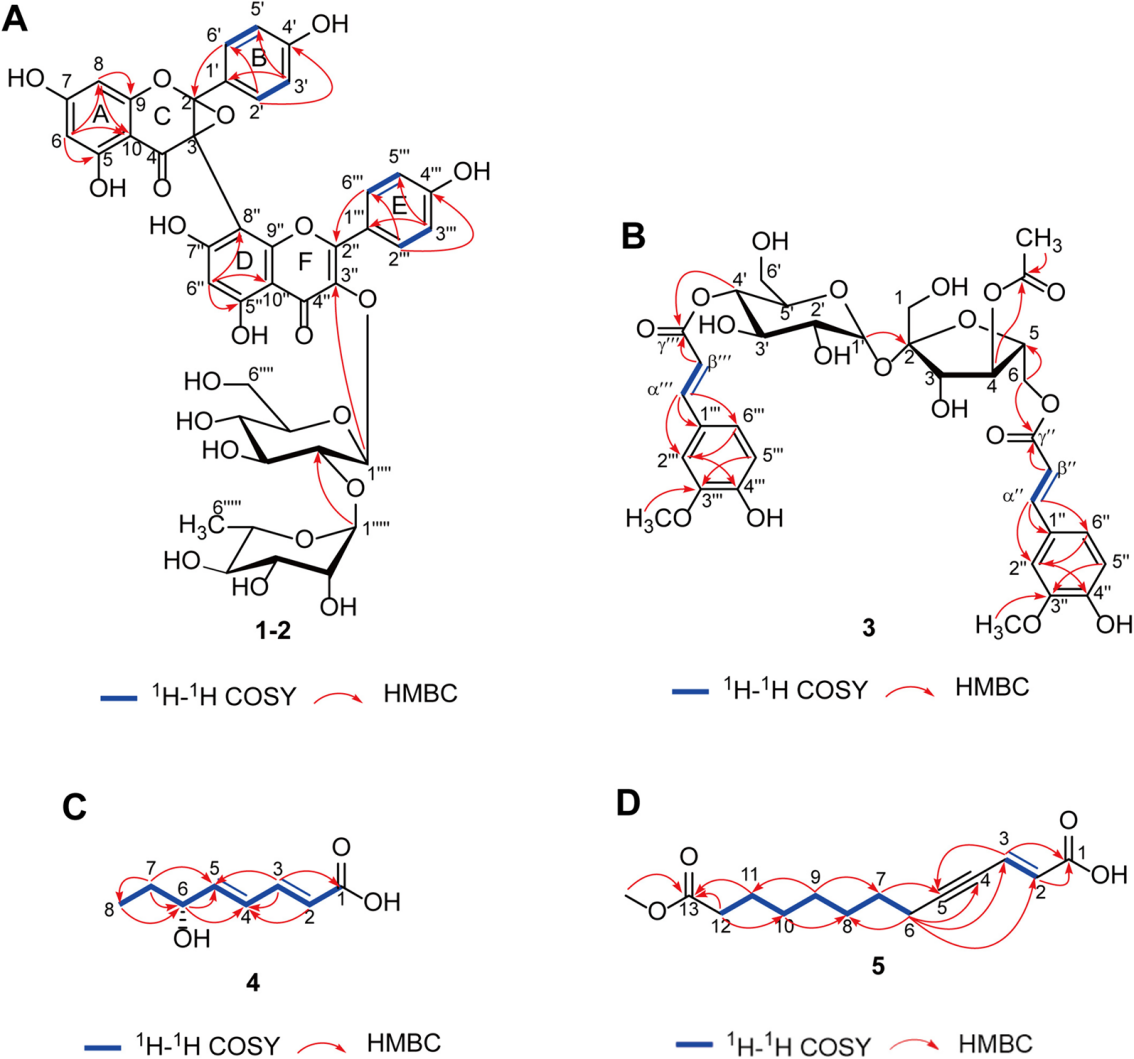
Key HMBC and ^1^H-^1^H COSY correlations. **A** compounds **1**–**2**. **B** compound** 3**. **C** compound** 4**. **D** compound** 5**

Compound **1** (thesiuside A) was obtained as a yellowish solid, and the molecular formula was deduced as C_42_H_38_O_21_ based on HRESIMS and the protonated molecular ion [M + H] ^+^ at *m/z* 879.1991 (calcd. for 879.1978). The ^1^H NMR spectrum (Table 1) displayed two groups of AA′BB′ aromatic spin system signals [ring B, *δ*_H_ 7.35 (2H, d, *J* = 8.9 Hz), 6.78 (2H, d, J = 8.9 Hz); ring E, *δ*_H_ 8.12 (2H, d, *J* = 9.0 Hz), 6.90 (2 H, d, J = 9.0 Hz)]. In addition, ring A was a tetrasubstituted aromatic ring [*δ*_H_ 5.96 (1H, d, *J* = 2.1 Hz), 5.90 (1H, d, *J* = 2.1 Hz)] and ring D was an 1,2,3,4,5-pentasubstituted aromatic ring [*δ*_H_ 6.57 (1H, s)]. Besides, two anomeric protons were identified at *δ*_H_ 5.74 (1H, d, *J* = 7.7 Hz) and 5.23 (1H, d, *J* = 1.9 Hz), and a series of proton signals on sugars were identified at *δ*_H_ 3–4, which revealed a *β*-glucopyranosyl moiety and an *α*-rhamnopyranosyl moiety. The ^13^C NMR spectrum (Table 1) exhibited a carbonyl signal (*δ*_C_ 192.5), an *α, β*-unsaturated carbonyl group (*δ*_C_ 179.6), two olefinic protons (*δ*_C_ 158.8, 134.7), 24 aromatic carbon signals. It was worth noting that the carbon resonance at *δ*_C_ 119.5 and *δ*_C_ 81.7 were ascribed to C-2 and C-3 bearing oxygen atoms. According to the molecular formula, C-2 and C-3 should form a ternary ring through one oxygen atom. In addition, further analyses of HMBC associations of H-2′ to C-2 and H-2′′′ to C-2′′ revealed that ring B and ring C were connected as well as ring D and ring E were connected. Thus, the relative configuration of **1** was proposed as a biflavone glycoside, which was formed by a structure of kaempferol (ring A, B and C), an epoxy flavanone (ring D, E and F) and two sugar substituents. And the C-3 in ring C and C-8′′ in ring D were the only possible connection mode of these two flavonoid structures. The 2D NMR data (HSQC, ^1^H-^1^H COSY and HMBC) of **1** showed the signal attribution of the *β*-glucopyranosyl moiety [*δ*_H_ 5.74 (1H, d, *J* = 7.7 Hz), 3.73 (1H, dd, *J* = 12.0, 2.1 Hz), 3.63 (1H, dd, *J* = 9.2, 7.7 Hz), 3.55 (1H, dd, *J* = 9.2, 8.7 Hz), 3.49 (1H, dd, *J* = 12.0, 5.9 Hz), 3.27 (1H, dd, *J* = 9.8, 8.7 Hz) and 3.23 (1H, ddd, *J* = 9.8, 5.9, 2.1 Hz), and *δ*_C_ 100.1, 79.7, 79.0, 71.9, 78.5 and 62.9] and the *α*-rhamnopyranosyl moiety [*δ*_H_ 5.23 (1H, d, *J* = 1.9 Hz), 3.98 (1H, dd, *J* = 3.4, 1.9 Hz), 3.98 (1H, overlap), 3.73 (1H, dd, *J* = 9.5, 3.4 Hz), 3.33 (1H, overlap) and 0.91 (3H, d, *J* = 6.2 Hz), and *δ*_C_ 102.5, 72.4, 72.3, 74.0, 69.9 and 17.5]. The HMBC cross-peak from H-1′′′′ to C-3′′ suggested the position of the *β*-glucopyranosyl moiety (Fig. 1A). It was further confirmed that *β*-glucopyranosyl moiety was linked at C-2′′′′ to *α*-rhamnopyranosyl moiety at C-1′′′′′ based on the HMBC spectrum.

The determination of the absolute configuration of compound **1** was elucidated via the ECD spectrum. The ECD spectrum (Additional file 1: Fig. S8) showed positive cotton effects for the π → π* transition at 320 nm indicating the presence of para substituted phenolic motif and a negative cotton effect for the n → π* transition at 288 nm proving the presence of ketone carbonyl group. The relative configuration of **1** was analogous to the structure of [(2*S*,3*S*)-2,3-epoxy-5,7,4′-trihydroxyflavanone] -(3→8)-kaempferol 3″-O-*β*-D-glucopyranoside, which has reported the ECD spectrum of its hydrolysate, and the ECD spectrum of **1** has similar cotton effects with its ECD spectrum [24]. Consequently, the absolute configuration of C-2 and C-3 was determined as *S*. Based on these results, compound **1** was proposed to be [(2*S*,3*S*)-2,3-epoxy-5,7,4′-trihydroxyflavanone]-(3→8)-kaempferol 3″-O-*α*-L-rhamnopyranosyl-(1→2)-*β*-D-glucopyranoside, and named as thesiuside A.

Compound **2** (thesiuside B) was isolated as a yellowish solid and its molecular formula was determined to be C_42_H_38_O_21_ by the HRESIMS, which exhibited the molecular ion [M + H] ^+^ peak at *m/z* 879.1991 (calcd. for 879.1978). Intriguingly, the 1D (^1^H NMR and ^13^C NMR) and 2D NMR (HSQC, ^1^H-^1^H COSY and HMBC) data of **2** (Table 1 and Fig. 1A) resembled those of **1**, which revealed that** 2** was a diastereomer of **1**. Their relative configurations were identical, and the only difference was the absolute configuration at positions C-2 and C-3. The ECD spectrum of **2** (Additional file 1: Fig. S15) and** 1** had opposite cotton effect at 320 nm and 288 nm, which suggests that the absolute configuration of **2** at C-2 and C-3 was *R*. Accordingly, the structure **2** was established as [(2*R*,3*R*)-2,3-epoxy-5,7,4′-trihydroxyflavanone]-(3 → 8)-kaempferol 3″-O-*α*-L-rhamnopyranosyl-(1 → 2)-*β*-D-glucopyranoside, and named as thesiuside B.

Compound **3** was purified as a colorless amorphous solid. The molecular formula was deduced as C_34_H_40_O_18_ by HRESIMS, which displayed the molecular ion [M − H] ^−^ at *m/z* 735.2159 (calcd. for 735.2142). The ^1^H NMR data (Table 2) showed characteristic signals for one oxygenated methyl group [*δ*_H_ 2.04 (3H, s)], three oxygenated methylenes [*δ*_H_ 4.48 (1H, d, *J* = 6.8 Hz) and 4.47 (1H, d, *J* = 3.7 Hz); 4.15 (1H, dd, *J* = 12.1, 5.5 Hz) and 4.13 (1H, dd, *J* = 12.1, 2.9 Hz); 3.72 (2H, s)], eight oxygenated methines [*δ*_H_ 5.47 (1H, d, *J* = 3.8 Hz), 4.93 (1H, dd, *J* = 10.2, 9.2 Hz), 4.40 (1H, ddd, *J* = 10.2, 5.5, 2.9 Hz), 4.19 (1H, d, *J* = 8.2 Hz), 4.14 (1H, overlap), 4.03 (1H, overlap), 4.02 (1H, dd, *J* = 9.7, 9.2 Hz), 3.58 (1H, dd, *J* = 9.7, 3.8 Hz)], four olefinic protons [*δ*_H_ 7.63 (1H, d, *J* = 16.0 Hz), 7.62(1H, d, *J* = 16.0 Hz), 6.39 (1H, d, *J* = 16.0 Hz), 6.33 (1H, d, *J* = 16.0 Hz)], which were two *trans* double bonds on the basis of coupling constants, two aromatic rings of ABX spin system [*δ*_H_ 7.13 (1H, d, *J* = 2.0 Hz), 7.03 (1H, dd, *J* = 8.2, 2.0 Hz) and 6.80 (1H, d, *J* = 8.2 Hz); 7.12 (1H, d, *J* = 2.0 Hz), 7.02 (1H, dd, *J* = 8.2, 2.0 Hz) and 6.78 (1H, d, *J* = 8.2 Hz)]. The ^13^C NMR and DEPT spectrum (Table 2) exhibited 34 carbon signals, corresponding to three ester carbonyl carbons (*δ*_C_ 172.7, 169.0, 168.3), sixteen sp^2^ carbons (*δ*_C_ 147.4, 147.1, 115.2, 115.2, 150.7, 150.7, 149.3, 149.3, 127.7, 127.6, 124.2, 124.1, 116.5, 116.5, 112.0, 111.8), three methyl carbons (*δ*_C_ 56.5, 56.5, 20.8) and twelve oxygenated carbons related to sugar groups (*δ*_C_ 105.9, 81.0, 78.9, 76.5, 66.4, 63.6; *δ*_C_ 93.5, 73.4, 72.6, 72.6, 69.9, 64.5). In addition, the 2D NMR data (HSQC, ^1^H-^1^H COSY and HMBC) suggested the existence of two identical *trans* ferulic acid structures and two sugars. Further analysis of the anomeric protons [*δ*_H_ 5.47 (1H, d, *J* = 3.8 Hz), 3.72 (1H, s)] and anomeric carbons (*δ*_C_ 93.5, 63.6) revealed that the structures of the two sugars were one *β*-D-fructose moiety and one *α*-D-glucose moiety, respectively. The HMBC cross-peak (Fig. 1B) from H-1′ to C-2 established the linkage of the two sugar moieties, indicating the presence of a sucrose moiety. After the glycohydrolysis experiments, it was determined that the sugar was sucrose analyzed by TLC and HPLC. The HMBC spectrum exhibited key correlations from H-6 to C-γ′′, from H-4′ to C-γ′′′ and from H-4 to carbonyl carbon (*δ*_C_ 172.7), which further revealed the connecting positions of two ferulic acids and one acetyl group. This compound was similar to compound helonioside B [25], with the main difference being the different connection positions of the *trans* ferulic acid groups and the acetyl group. Therefore, **3** was established as 4-acetyl-6, 4′-diferuloylsucrose.

Compound **4** was obtained as a yellowish oil. HRESIMS measurements of this compound showed a [M − H] ^−^ ion peak at *m/z* 155.0706 and the molecular formula was assigned as C_8_H_12_O_3_ (calcd. for C_8_H_11_O_3_, 155.0714), deducing three degrees of unsaturation. The ^1^H NMR spectrum (Table 3) indicated that **4** contained one methyl group [*δ*_H_ 0.94 (3H, t, *J* = 7.4 Hz)], one methylene [*δ*_H_ 1.56 (2H, m)], one oxygenated methine [*δ*_H_ 4.08 (1H, q, *J* = 6.1)] and four olefinic protons [*δ*_H_ 7.22 (1H, dd, *J* = 15.4, 11.0 Hz), 6.39 (1H, dd, *J* = 15.2, 11.0 Hz), 6.10 (1H, dd, *J* = 15.2, 11.0 Hz), and 5.89 (1H, d, *J* = 15.4 Hz)]. The ^13^C NMR data (Table 3) of **4** confirmed the presence of 22 carbon resonances, including one methyl carbon (*δ*_C_ 10.1), one methylene carbon (*δ*_C_ 30.9), one oxygenated methine carbon (*δ*_C_ 74.0), four olefinic carbons (*δ*_C_ 145.8, 144.8, 128.8 and 124.0) and one carboxylic carbon (*δ*_C_ 171.8). ^1^H-^1^H COSY correlations (Fig. 1C) of H-2/H-3/H-4/H-5/H-6/H-7/H-8 and the HMBC cross-peaks (Fig. 1C) of H-2/C-4; H-3/C-1, C-3; H-6/C-4; H-7/C-5 and H-8/C-6 proved that the compound bears a long-chain fatty acid structure. Furthermore, the *E* configurations of the C-2/C-3 and C-4/C-5 double bonds were determined by the coupling constants, which also explained the presence of an *α*, *β*, *γ*, *δ*-unsaturated carbonyl moiety. The absolute configuration of the hydroxyl group at C-6 was assigned to be *R* by measuring the specific rotation of **4** ([α]_D_^20^ -3.3), which is opposite to that of the similar compound previously reported [26]. Finally, compound **4** was elucidated as (2*E*,4*E*,6*R*)-6-hydroxy-2,4-oxtadienoic acid.

Compound **5** was isolated as a yellow oil. It had a molecular formula of C_14_H_20_O_4_ inferred from the [M − H] ^−^ ion peak at *m/z* 251.1279 (calcd. for 251.1289), which was deduced from the HRESIMS spectrum, and it required three degrees of unsaturation. The ^1^H NMR spectrum (Table 3) gave signals for one methyl group [*δ*_H_ 3.65 (3H, s)], seven methylenes [*δ*_H_ 2.40 (2H, td, *J* = 7.0, 2.0 Hz), 2.32 (2H, t, *J* = 7.4 Hz), 1.62 (2H, m), 1.56 (2H, m), 1.42 (2H, m), 1.35 (2H, m), 1.35 (2H, m)] and two olefinic protons [*δ*_H_ 6.69 (1H, dt, *J* = 15.9, 2.0 Hz), 6.10 (1H, d, *J* = 15.9 Hz)]. Its ^13^C NMR and DEPT spectrum (Table 3) suggested the presence of one methyl carbon (*δ*_C_ 52.0), seven methylene carbons (*δ*_C_ 34.7, 30.0, 29.8, 29.7, 29.4, 25.9, 20.2), two olefinic carbons (*δ*_C_ 131.5, 126.8), two carbonyl carbons (*δ*_C_ 176.0, 169.7) and two acetylenic carbons (*δ*_C_ 101.2, 78.8). A long methylene chain unit was established by the ^1^H-^1^H COSY spectrum (Fig. 1D), which showed the correlations at H-6/H-7/H-8/H-9/H-10/H-11/H-12. The HMBC cross-peaks (Fig. 1D) of H-3/C-1, C-5; H-2/C-1; H-6/C-2, C-3, C-4, and H-7/C-5 confirmed that one end of the acetylenic bond was connected to the fat chain and the other to the *α*, *β*-unsaturated carbonyl group, which could also be identified by the ^1^H-^1^H COSY cross-peaks from H-3 to H-6. The existence and position of the methyl ester group were supported by the HMBC data, which indicated the cross-peaks from methyl hydrogen to C-13 and from H-11 to C-13. Furthermore, the coupling constants between H-2 and H-3 (*J* = 15.9 Hz) suggested the C-2/C-3 double bonds were *E* geometry. The structure of compound **5** was similar to the known compound anacolosine, the main difference was the presence of methyl ester unit (*δ*_H_ 3.65, *δ*_C_ 52.0) in compound **5** [27]. Therefore, compound **5** was named as (*E*)-13-methoxy-13-oxotridec-2-en-4-ynoic acid.

**Fig. 2:**
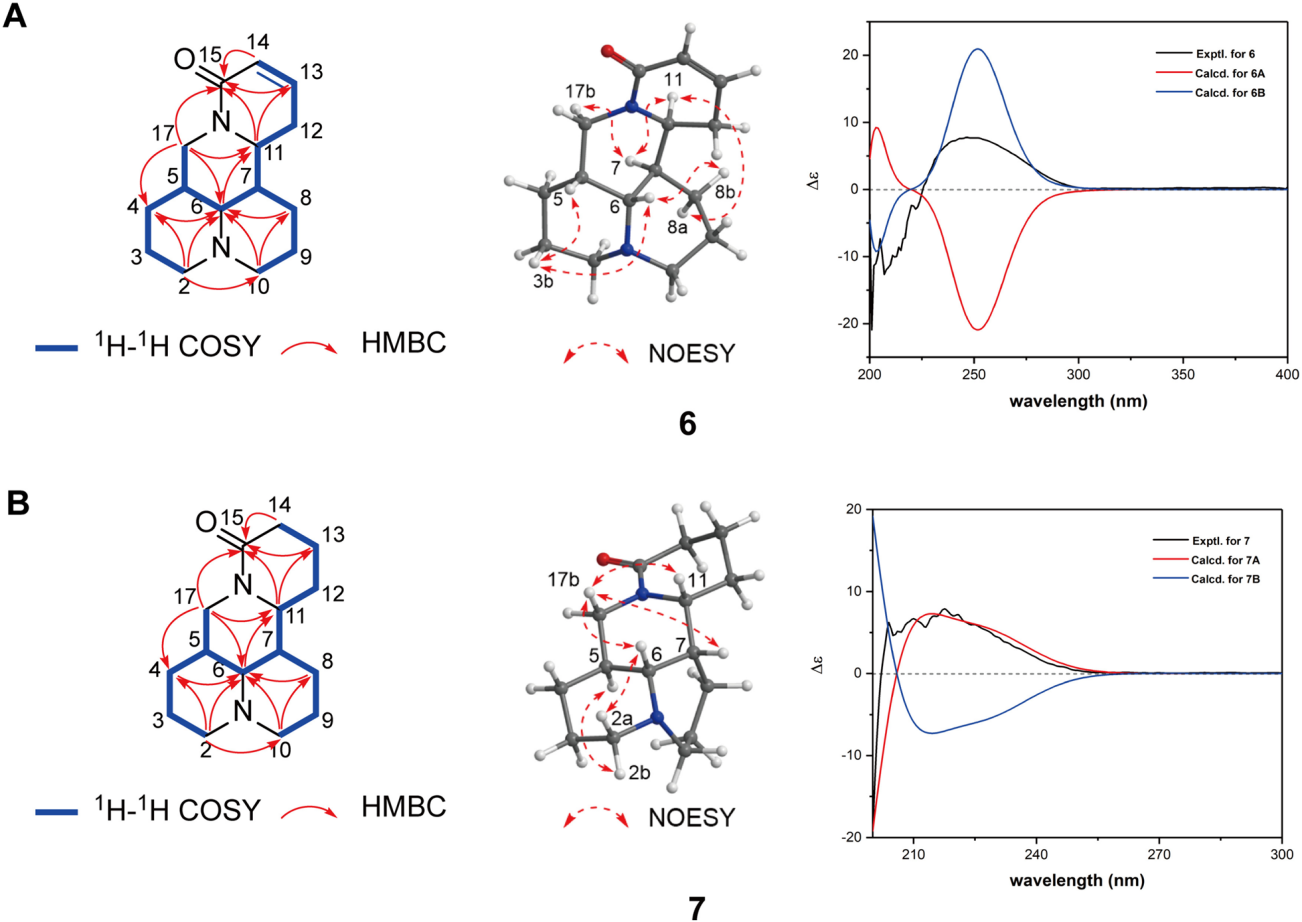
**A** Key HMBC, ^1^H-^1^H COSY correlations and key NOESY correlations, and experimental and calculated ECD spectra of compound **6**; **B** Key HMBC, ^1^H-^1^H COSY correlations and key NOESY correlations, and experimental and calculated ECD spectra of compound **7**

Compound **6** was obtained as a colorless oil. The [M + H] ^+^ ion was observed at 247.1805 *m/z* (calcd. for 247.1805) by HRESIMS and the molecular formula of **6** was deduced as C_15_H_22_N_2_O with 6 degrees of unsaturation. The ^1^H NMR spectrum (Table 4) and HSQC spectrum revealed the presence of two olefinic protons [*δ*_H_ 6.64 (1H, ddd, *J* = 9.8, 4.7, 3.8 Hz) and 5.84 (1H, dt, *J* = 9.8, 2.0 Hz)], eight methylenes [*δ*_H_ 3.46 (1H, overlap) and 2.89 (1H, overlap), 1.97 (1H, overlap) and 1.72 (1H, overlap), 1.85 (1H, m) and 1.80 (1H, overlap), 2.01 (1H, overlap) and 1.72 (1H, overlap), 2.07 (1H, overlap) and 1.72 (1H, overlap), 3.46 (1H, overlap) and 2.89 (1H, overlap), 2.79 (1H, m) and 2.34 (1H, m) as well as 4.25 (1H, dd, *J* = 14.0, 5.0 Hz) and 3.23 (1H, dd, *J* = 14.0, 12.9 Hz)], and four methines [*δ*_H_ 4.13 (1H, ddd, *J* = 11.8, 8.4, 7.3 Hz), 3.39 (1H, t, *J* = 3.2 Hz), 2.11, (1H, overlap) and 2.07, (1H, overlap)]. The analysis of ^13^C NMR and DEPT spectrum (Table 4) showed 15 carbon resonances, including eight methylene carbons (*δ*_C_ 57.0, 56.9, 42.0, 28.0, 26.8, 25.2, 20.2, 19.8), four methines (*δ*_C_ 64.6, 52.0, 41.2, 34.8), two olefinic carbons (*δ*_C_ 140.7, 124.1) and one carbonyl carbon (*δ*_C_ 167.6). The ^1^H-^1^H COSY cross-peaks of H-14/H-13/H_2_-12/H-11, together with the HMBC correlations (Fig. 2A) from H-14 to C-15, from H-13 to C-15 and from H-11 to C-13/C-15 established the location of the *α*, *β* unsaturated ketone. The ^1^H-^1^H COSY cross-peaks of H-11/H-7/H-6/H-5/H_2_-17, H-7/H_2_-8/H_2_-9/H_2_-10 and H-5/H_2_-4/H_2_-3/H_2_-2 together with the HMBC correlations from H-11 to C-15, from H-17 to C-15, from H-17 to C-11, from H_2_-2 to C-6, from H_2_-10 to C-6 and from H_2_-2 to C-10 revealed that the skeleton of **6** was a matrine-type alkaloid. The relative configuration of **6** was deduced by the NOESY spectrum (Fig. 2A), where correlations of H-17b/H-7 and H-11, H-6/H-8b as well as H-11/H-8a exhibited the H-7 and H-11 were *β*-orientations. The cross-peaks of H-3b/H-5 and H-6 suggested the *α*-orientations of H-5 and H-6.

The C-5, C-6, C-7, and C-11 of the compound were chiral carbon atoms, and there may be two absolute configurations of the compound, namely enantiomers **6A** (5*R*, 6*R*, 7*R*, 11*S*) and **6B** (5*S*, 6*S*, 7*S*, 11*R*), respectively. The ECD spectra of the two configurations were obtained by the method of ECD calculations. It was found that the calculated ECD curves of **6B** (5*S*, 6*S*, 7*S*, 11*R*) were basically consistent with those of compound **6** measured by experiments (Fig. 2A). Thus, its absolute configuration was determined as 5*S*, 6*S*, 7*S*, 11*R*. Compound **6** was established as a new structure and named as thesiumine A.

Compound **7** was obtained as a colorless oil. The [M + H] ^+^ ion was observed at 249.1960 m*/z* (calcd. for 249.1961) by HRESIMS and the molecular formula of **7** was deduced as C_15_H_24_N_2_O with 5 degrees of unsaturation. The significant difference between the ^1^H NMR and ^13^C NMR data (Table 4) of **7** and those of **6** was the absence of a double bond. The NOESY correlations (Fig. 2B) of H-17b/H-6, H-7 and H-11 exhibited the H-11, H-7 and H-6 were *β*-orientations. The cross-peaks of H-2b/H-6 and H-2a/H-5 indicated the *α*-orientations of H-5. The absolute configuration was also determined by ECD calculations with the same method of compound **6**. The experimental ECD spectrum of compound **7** was compared with the calculated ECD spectrum (Fig. 2B and Additional file 1: Fig. S51) of compound **7A** (5*S*, 6*R*, 7*S*, 11*R*) and **7B** (5*R*, 6*S*, 7*R*, 11*S*). The results showed that the ECD curves of **7A** (5*S*, 6*R*, 7*S*, 11*R*) configuration were in good agreement with those of compound **7**. Therefore, its absolute configuration was determined as 5*S*, 6*R*, 7*S*, 11*R*. Compound **7**, which was an enantiomer of tetrahydroneosophoramine [28], was identified as a new compound named thesiumine B.

**Fig. 3:**
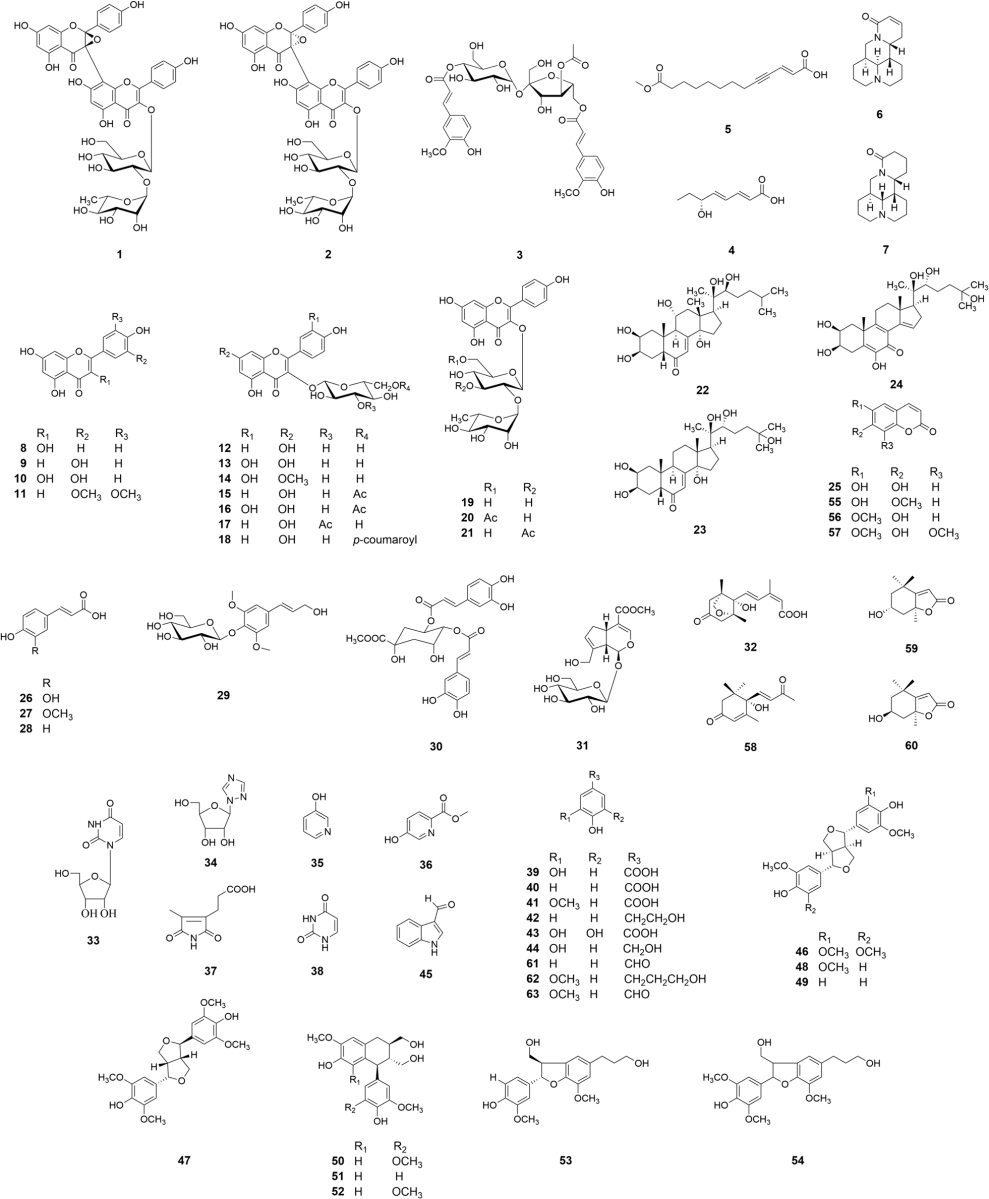
Chemical structures of compounds **1**–**63** isolated from the *T. chinense*

The other 56 known compounds (Fig. 3) were identified by comparing their spectroscopic data with those reported in the literature (Additional file 1: Table S2), including kaempferol (**8**), luteolin (**9**), quercetin (**10**), tricin (**11**), astragalin (**12**), isoquercitrin (**13**), rhamnetin-3-*O*-*β*-D-glucopyranoside (**14**), kaempferol-3-*O*-(6′′-*O*-acetyl)-*β*-D-glucopyranoside (**15**), quercetin-3-*O*-(6′′-O-acetyl)-*β*-D-glucopyranoside (**16**), kaempferol-3-*O*-(3′′-*O*-acetyl)-*β*-D-glucopyranoside (**17**), tiliroside (**18**), kaempferol-3-*O*-glucorhamnoside (**19**), kaempferol-3-*O*-*α*-L-rhamnopyranosyl-(1 → 2)-[6-*O*-acetyl]-*β*-D-glucopyranoside (**20**), kaempferol-3-*O*-*α*-L-rhamnopyranosyl-(1 → 2)-[3-*O*-acetyl]-*β*-D-glucopyranoside (**21**), ajugasterone C (**22**), 20-hydroxyecdysone (**23**), calonysterone (**24**), esculetin (**25**), caffeic acid (**26**), (*E*)-ferulic acid (**27**), (*E*)-*p*-coumaric acid (**28**), syringin (**29**), 4,5-di-*O*-caffeoylquinic acid 1-methyl ether (**30**), geniposide (**31**), phaseic acid (**32**), uridine (**33**), 1-(*β*-D-ribofuranosyl)-1*H*-1,2,4-triazone (**34**), 3-hydroxypyridine (**35**), methyl-5-hydroxypyridine-2-carboxlate (**36**), isohematinic acid (**37**), uracil (**38**), protocatechuic acid (**39**), *p*-hydroxybenzoic acid (**40**), vanillic acid (**41**), *p*-hydroxyphenethyl alcohol (**42**), gallic acid (**43**), 3,4-dihydroxybenzyl alcohol (**44**), 1*H*-indole-3-carboxaldehyde (**45**), ( +)-syringaresinol (**46**), lirioresionol (**47**), ( +)-medioresinol (**48**), ( +)-pinoresinol (**49**), 5-methoxy-( +)-isolariciresinol (**50**), ( +)-isolariciresinol (**51**), ( +)-lyoniresinol (**52**), (7*S*, 8*R*)-dihydrodehydrodiconiferyl alcohol (**53**), 5-methoxydehydroconiferyl alcohol (**54**), isoscopoletin (**55**), scopoletin (**56**), isofraxidin (**57**), ( +)-dehydrovomifoliol (**58**), ( −)-loliolide (**59**), ( +)-isololiolide (**60**), *p*-hydroxyacetophenone (**61**), dihydroconiferylalcohol (**62**), acetovanillone (**63**). Notably, forty-seven previously unseparated compounds (**10**–**11**, **13**–**18**, **20**–**24**, **26**–**27**, **29**–**39**, **41**–**58**, **60**, and **62**–**63**) were discovered from the plant in addition to the compounds **1**–**7**.

### Inhibition of NO production by isolated compounds

The in vitro anti-inflammatory activity of isolated compounds was evaluated by detecting NO level in LPS-induced RAW 264.7 macrophages. Cytotoxicity of the compounds was determined by MTT assay to confirm that the reduction in NO production was not due to inhibition of cell proliferation.

**Fig. 4:**
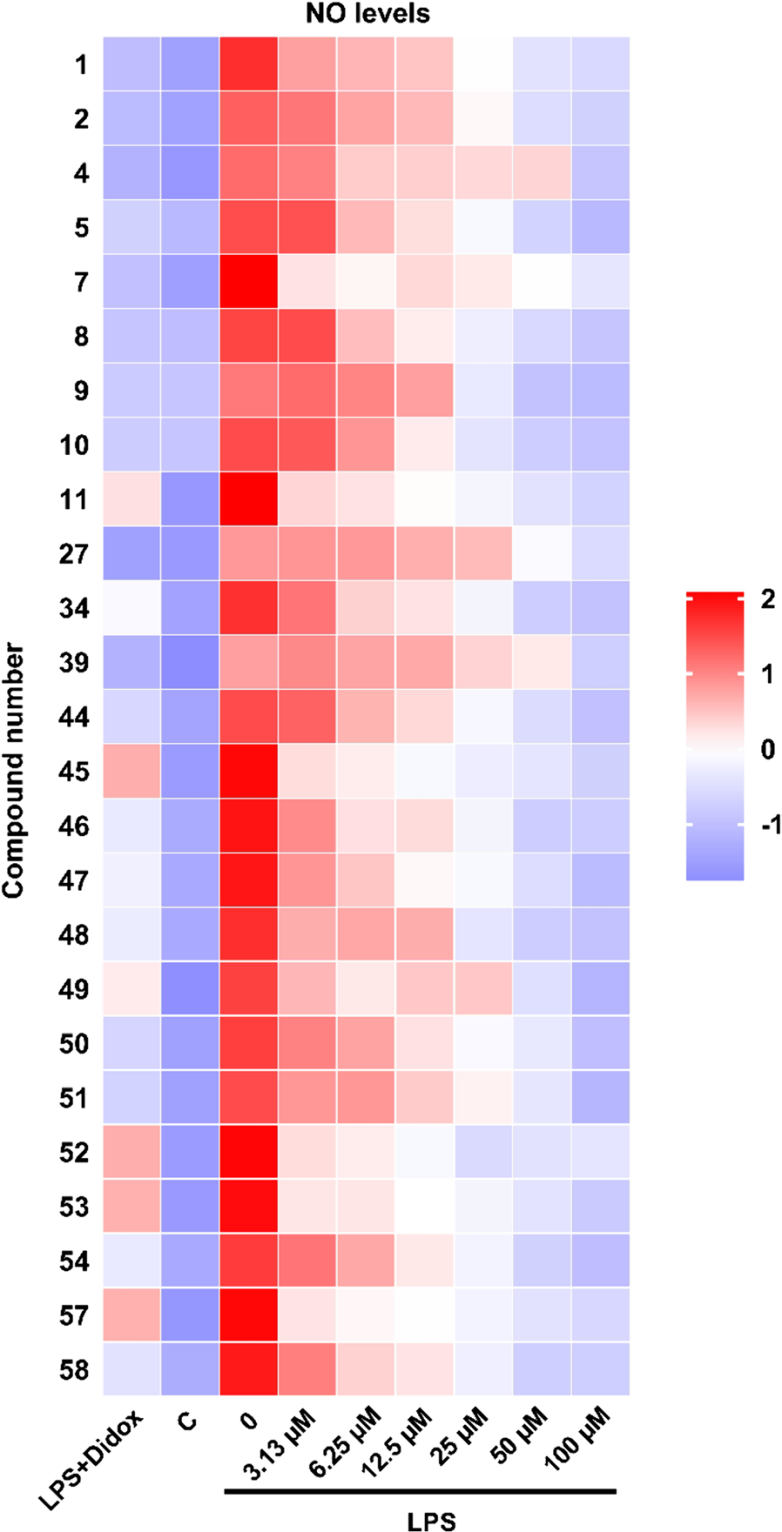
Heat map analysis of inhibitory activity of compound on LPS-induced NO production in RAW 264.7 cells. NO level was measured after treatment with compounds at indicated doses along with LPS (1 μg/mL) for 24 h. The data were normalized using Z-score (https://www.omicshare.com/tools). The color of the spots represents the level of NO production in each group, with red indicating a higher level and blue indicating a lower level. C: control group

New compounds **1**, **2**, **4** and **5** displayed NO production inhibition activity. Flavonoid aglycones **8**–**11** and fatty acid **5** displayed strong anti-inflammatory activity and their MIRs either exceeded or were equal to that of DIDOX (Fig. 4). Flavonoid glycosides **1** and **2**, fatty acid **4**, alkaloids **7** and **34**, phenylpropionic acid **27**, simple aromatics **39**, lignans **44–54**, coumarin **57** and terpenoid **58** demonstrated moderate anti-inflammatory activity. It was worth mentioning that although flavonoid glycosides were the main constituents of *T. chinense*, compounds **12**–**21** exhibited no inhibitory activity of NO production.

### Targets of *T. chinense* against lung inflammation based on network pharmacology analysis

**Fig. 5:**
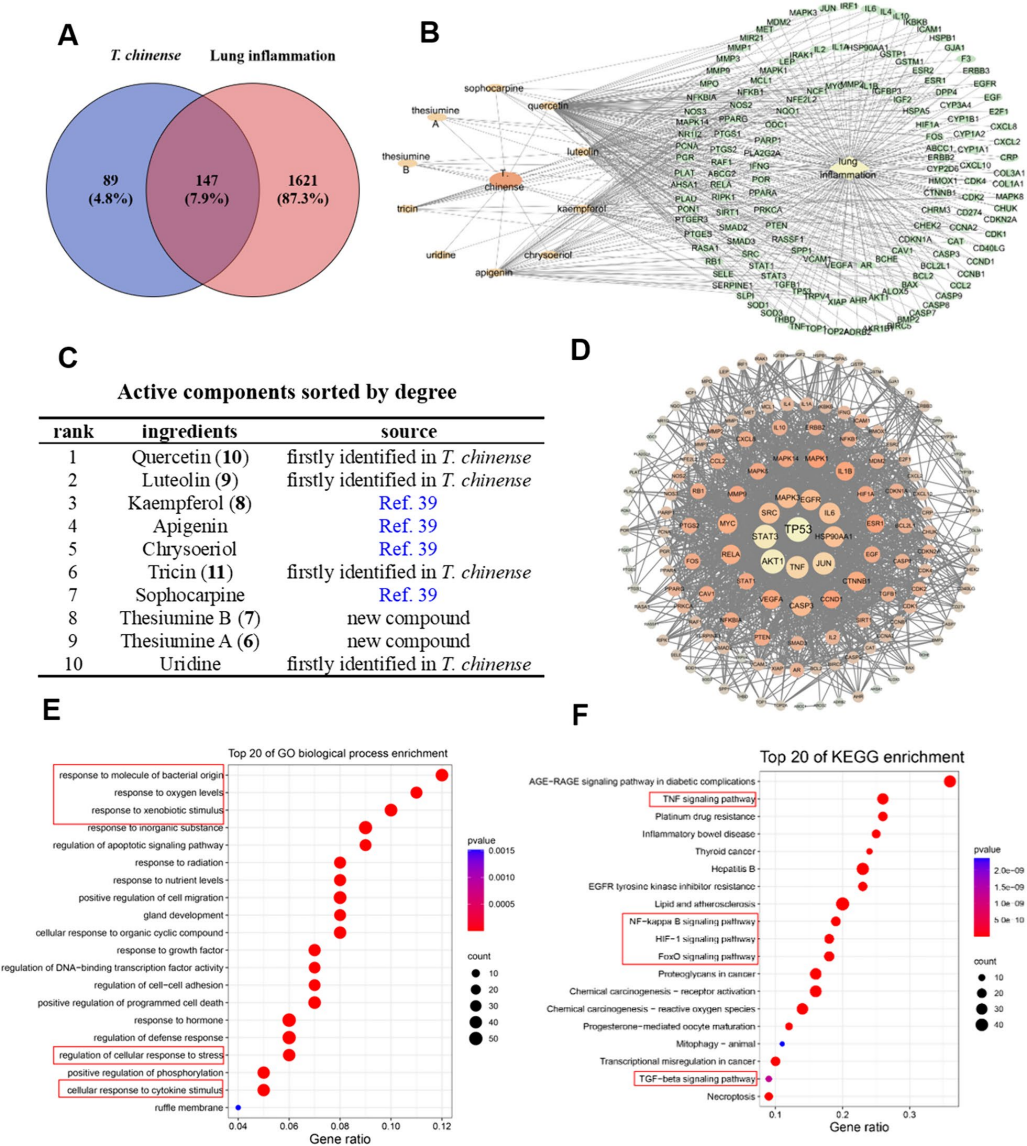
Results of network pharmacology prediction of *T. chinense* for lung inflammation. **A** Venn diagram of lung inflammation target and *T. chinense* target. **B** The “constituent-target-disease” network of *T. chinense*. The pink oval represents *T. chinense*, the green oval represents the active constituents in *T. chinense*, yellow oval represents predicted target, and blue diamond represents lung inflammation. **C** Active components sorted by degree. **D** Common target protein interaction network diagram. **E** GO biological process enrichment analysis of the potential targets of *T. chinense* against inflammation. **F** KEGG signaling pathway enrichment analysis on the potential targets of *T. chinense* against inflammation

For further exploration of the potential targets of *T. chinense* against lung inflammation, we conducted a network pharmacology analysis of *T. chinense* against lung inflammation. A total of 88 constituents in *T. chinense* were obtained from literature reviews and our identified constituents in this article, and 10 active constituents were obtained after screening by Swiss target ADME. 236 drug targets and 1768 disease targets were collected from the HERB, TCMSP, and Genecards databases. We matched targets of the ingredients and the disease, 147 common targets were obtained and shown with a venn diagram (Fig. 5A; Additional file 1: Table S4). To better demonstrate the connection between drugs and disease targets, a *T. chinense*-constituents-targets-lung inflammation network diagram was constructed (Fig. 5B). The active ingredients were sorted by degree and displayed in Fig. 5C, and of which, five constituents (**6–7** and **9–11**) in the top ten active constituents were new compounds or firstly isolated from *T. chinense*. Protein–protein interaction (PPI) network was constructed to further explore the protein–protein interaction relationship between common targets. The key proteins for *T. chinense* treating lung inflammation included TP53, AKT1, STAT3, TNF, JUN, IL6, HSP90AA1, SRC, MAPK3, EGFR (Fig. 5D). Gene ontology (GO) enrichment analysis demonstrated that *T. chinense* may intervene lung inflammation through participating in inflammation-related biological processes containing response to molecule of bacterial origin, response to xenobiotic stimulus, regulation of defense response, response to oxygen levels, regulation of cellular response to stress, and cellular response to cytokine stimulus (Fig. 5E). KEGG enrichment analysis showed that *T. chinense* intervened lung inflammation via regulating TNF signaling pathway, FoXO signaling pathway, NF-κB signaling pathway, HIF-1 signaling pathway, TGF-β signaling pathway, and other inflammation-related pathways (Fig. 5F). All these data indicated the enormous potential of *T. chinense* therapy for lung inflammation and potential therapeutic targets and mechanisms.

### The constituents, extract and preparation of *T. chinense* attenuate LPS-induced ALI in mice

Based on network pharmacology analysis, flavonoids and their glycosides play a crucial role in treating lung inflammation by *T*. *chinense* and Bairui Granules. The predominant and representative constituents were flavonoid glycosides KN (Bairuisu I, **19**) and AG (Bairuisu II, **12**) with contents of 15.072 mg/g and 8.014 mg/g in *T. chinense*, and 4.23 mg/g and 1.87 mg/g in Bairui Granules. Although they did not rank among the top ten most active constituents, their common glycoside, kaempferol, was predicted by network pharmacology analysis. In addition, **12** and **19** did not display an inhibitory effect against NO production. Thus, the two constituents (**19** and **12**) were further evaluated for their anti-inflammatory activity in vivo. Moreover, KF (Bairuisu III, **8**) with potent inhibition on NO production, as well as BG and CE have also been determined.

**Fig. 6:**
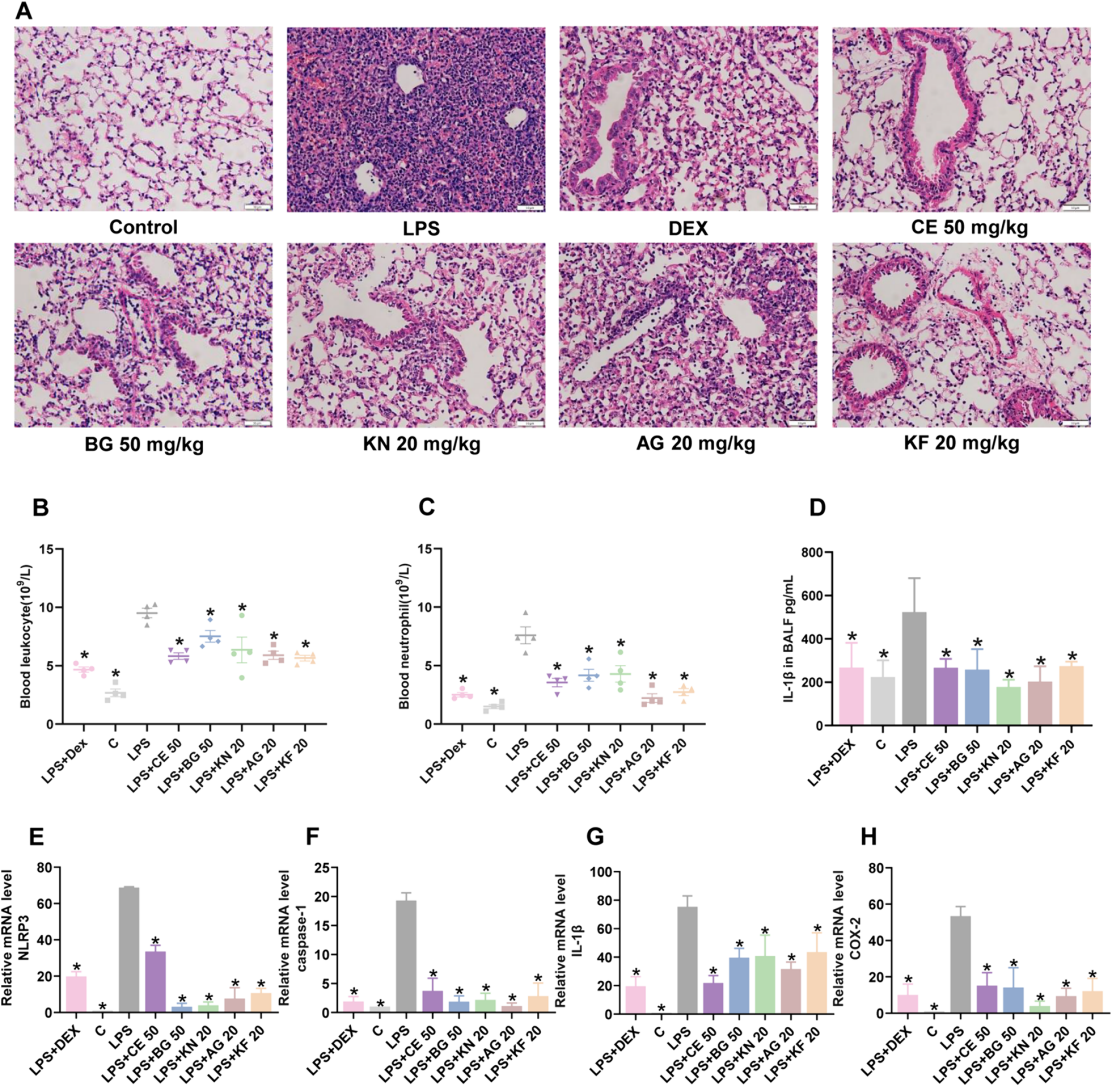
Effects of *T. chinense* on lung inflammation induced by LPS. **A** H&E staining of lung tissue sections. Scale bar, 50 µm. **B**, **C** Peripheral blood leukocyte and neutrophil count. **D** The level of IL-1β in BALF.** E**–**H** Relative mRNA level of NLRP3, caspase-1, IL-1β and COX-2. Data were expressed as mean ± SD (n = 3), * p < 0.05 as compared to LPS group

As displayed in Fig. 6A, LPS exposure led to the persistent infiltration of inflammatory cells, accumulation of neutrophils in the alveolar and interstitial space, the thickened alveolar and airway walls. While, these symptoms of inflammation were obviously alleviated after KN (**19**), AG (**12**), KF (**8**), BG and CE. Furthermore, compared with the control group, the count of leukocytes and neutrophils in peripheral blood increased significantly in LPS-treated group (Fig. 6B-C). And the number of these inflammation-related cells was reduced in mice treated by KN (**19**), AG (**12**), KF (**8**), BG and CE. Abnormal activation of NOD-like receptor protein 3 (NLRP3) inflammasome could lead to the high expression of caspase-1 and the secretion of IL-1β, leading to the occurrence of inflammatory diseases [29]. Acute inflammation could lead to high expression of cyclooxygenase-2 (COX-2) and damage tissues [30]. Thus, we measured the levels of the inflammatory cytokine IL-1β in the BALF using ELISA assay and the mRNA levels of NLRP3, caspase-1, IL-1β and COX-2 using RT-PCR. As depicted in Fig. 6D, treatment with KN (**19**), AG (**12**), KF (**8**), BG and CE blocked the LPS-stimulated increase of IL-1β in BALF. Similarly, they significantly inhibited the LPS-stimulated upregulation of mRNA levels of NLRP3, caspase-1, IL-1β and COX-2 in the lung tissues (Fig. 6E-H). These data suggested that KN (**19**), AG (**12**), KF (**8**), BG and CE were capable of alleviating lung inflammation. KN (**19**), AG (**12**) and KF (**8**) are responsible for the traditional and clinical application of *Thesium chinense* Turcz. and Bairui Granules against inflammation-related diseases. Nevertheless, there is a deficiency of established quality control standards and markers for *T. chinense*. It is proposed that KN (**19**) and AG (**12**) could serve as foundational elements to enhance quality standards or act as markers for assessing the quality of *T. chinense* and its preparations.

### Safety

#### Clinical observations and body weight including food intake.

In the subacute toxicity assessment, we conducted precise and systematic observations to detect potential signs of toxicity. These observations were conducted at specific time intervals, including 0, 30 min, 1, 3 and 6 h, followed by daily evaluations over a 28-day period. Notably, no fatalities or adverse indicators of toxicity, such as emesis, lethargy, paw jerking, and cutaneous lesions, were observed across all administered doses when compared to the normal control group (Table 5). Furthermore, despite the observed progressive increase in body weight throughout the experiment, we found no statistically significant differences in either body weight or food intake among the experimental groups (Additional file 1: Fig. S12A-B).

**Table 5:** Effects of Control, CE, BG-L and BG-H on physical and behavioral parameters in mice during a 28-day subacute toxicity study

Parameter	Control	CE	BG-L	BG-H
Survival rate	100%	100%	100%	100%
Evident anomalies	Not observed	Not observed	Not observed	Not observed
Respiratory rate	Normal	Normal	Normal	Normal
Vomitus	Not observed	Not observed	Not observed	Not observed
Lethargy	Not observed	Not observed	Not observed	Not observed
Micturition	Normal	Normal	Normal	Normal
Fecal coloration	Normal	Normal	Normal	Normal
Diarrhea	Not observed	Not observed	Not observed	Not observed
Mucoid stool	Not observed	Not observed	Not observed	Not observed
Eye colour	Normal	Normal	Normal	Normal
Skin colour	Normal	Normal	Normal	Normal
Cutaneous lesions	Not observed	Not observed	Not observed	Not observed
Paw jerking	Not observed	Not observed	Not observed	Not observed
Paw licking	Not observed	Not observed	Not observed	Not observed
Paw biting	Not observed	Not observed	Not observed	Not observed

#### Organ coefficients and morphological examination

**Fig. 7:**
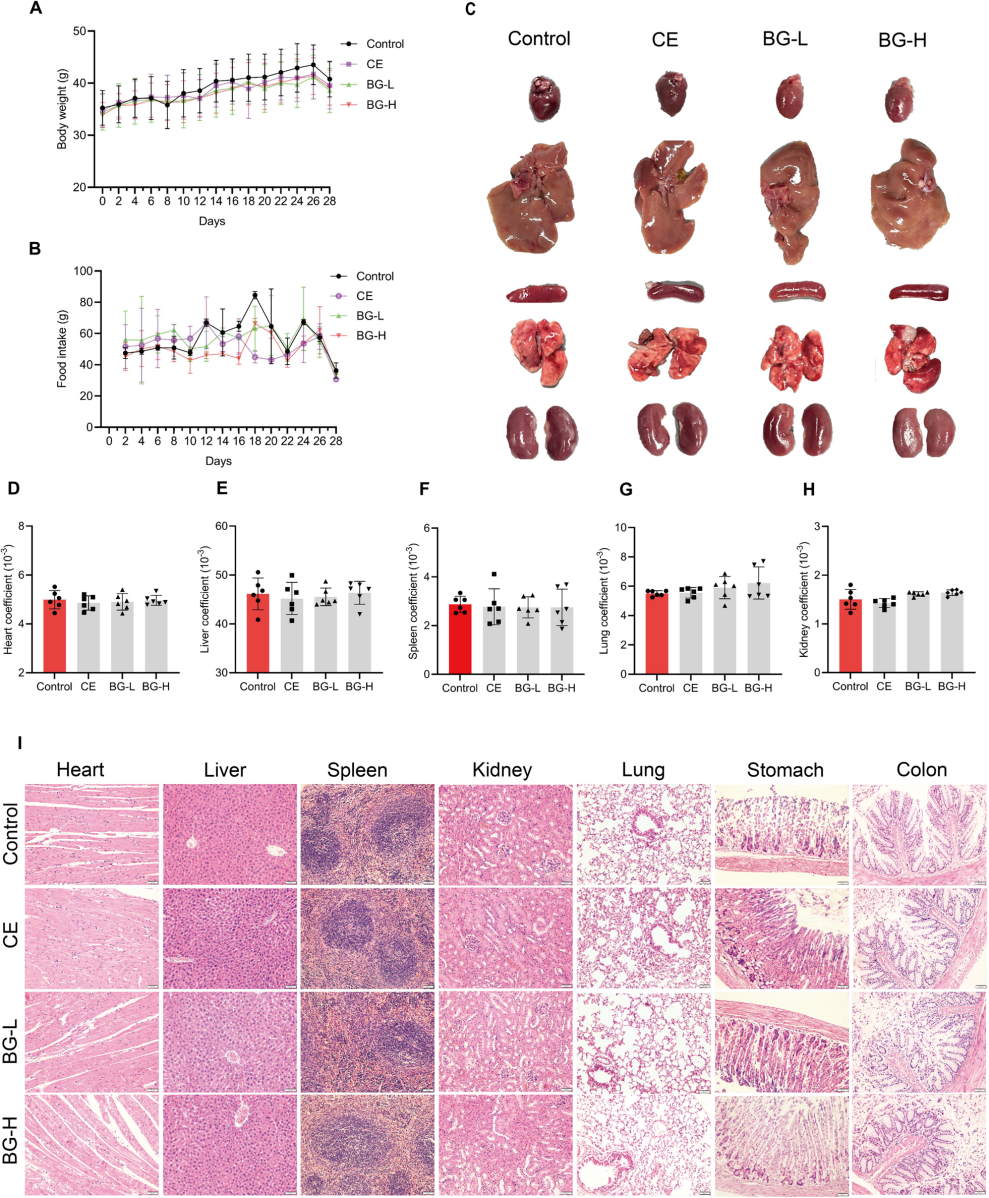
Effects of Control, CE, BG-L, BG-H on mice growth and organ characteristics in a 28-day subacute toxicity study. **A** Body weight. **B** Food intake. **C** The gross appearance of heart, liver, spleen, lung and kidney. **D**–**H** Organ coefficients of heart, liver, spleen, lung and kidney. **I** H&E staining of heart, liver, spleen, lung, stomach and colon tissue sections. Scale bar, 50 µm. Data are illustrated as mean ± SD (n = 6); * p < 0.05 as compared to the control group

Hearts, livers, lungs, kidneys and spleens were dissected, rinsed in saline, and then placed on a blank background under the white light for the photographic documentation. The results demonstrated the structural integrity and healthy appearance of the organs across all four groups of mice, with no significant differences observed (Fig. 7C). The organ coefficient, calculated as the ratio of organ mass to body weight, is a commonly employed parameter in toxicology experiments. In this study, there were no statistically significant differences in organ coefficients among the control group, CE, BG-L and BG-H group (Fig. 7D-G).

#### Histopathological assessment

Significant histological changes were not observed in the control and treatment groups.

Heart: The microscopic analysis of the heart in all treatment groups displayed normal architecture of the cardiac muscle fibers with intact length and regular cell striation and nuclei and was bereft of significant cellular infiltration or degeneration.

Liver: The tissue slices displayed intact hepatic parenchyma including hepatocytes, central vein and portal triad. No significant mixed macro and micro vesicular steatosis, vacuolated hepatocytes or any kind of infiltration was observed in the liver tissue (hepatocytes). Hepatocytes were organized in cords with intact cellular and nucleus borders.

Lung: All treated groups showed normal lung architecture. No significant damage (such as granulomas, increased alveolar cell wall, inflammatory cells) was observed in the lung tissue of any treated group rats. The section showed an intact alveolar membrane.

Spleen: No significant damage was observed in the spleen tissue of any treated group. The graph showed the normal white and red pulp areas with no cell breakage in the splenic parenchyma.

Kidney: In all treatment groups, the kidneys exhibited tightly arranged and well-organized glomerular and tubular structures.

Stomach: No significant damage (such as focal necrosis, mucus wall disruption, extensive congestion in the mucosa and haemorrhagic bands) was observed in the inner lining of stomach of any treated group. The inner mucus membrane was completely remained intact.

Colon: No significant damage (villi atropy, inflammation, superficial erosion and crypt hyperplasia) was observed in the tissues of small intestine of any treated groups rats. The histology result displays no abnormalities in the microscopic structure of small intestine.

#### Blood biochemistry and routine blood indicators

Blood supernatants were analyzed using a blood biochemistry analyzer, which revealed no statistically significant differences in cardiac function parameters, including CK and LDH, between the treatment and control groups (Additional file 1: Fig. S52A-D). Similarly, no statistically significant differences were observed in TBA, ALP, ALT and AST, which serve as indicators of liver function (Additional file 1: Fig. S52C-F). There is no significant difference in creatinine and urea nitrogen levels, which are indicative of renal function (Additional file 1: Fig. S52G-H).

The results of the blood routine analysis are presented in Additional file 1: Fig. S52. In this analysis, there were no statistically significant differences observed between the experimental group and the control group in various indicators used for assessing infection and immune function, such as white blood cell count, red blood cell count, lymphs, eosinophils, and neut/gran. Furthermore, parameters such as hemoglobin, hematocrit, mean corpuscular volume, mean platelet volume, mean corpuscular hemoglobin, mean corpuscular hemoglobin concentration and platelet count, which are indicative of anemia, blood viscosity, and platelet function, also showed no significant differences. In summary, the administration of CE, BG-L and BG-H did not have significant impacts on the blood and serum biochemical indices of the mice.

## Discussion

Pneumonia is the principal cause of ALI/ARDS, which can be brought on by gram-negative or positive bacteria, viruses, and other exogenous hazardous substances [31]. ALI is characterized by pulmonary inflammation, accompanied by a systemic “cytokine storm”, which simultaneously produces damage to the epithelium and endothelium, leading to the rupture of the alveolar capillary barrier, pulmonary edema and neutrophil infiltration, which can further develop into ARDS [32–34]. Inflammatory responses, such as the recruitment of neutrophils and the generation of proinflammatory cytokines IL-1β, are frequently present in ALI [4]. High level of proinflammatory cytokines, including COX-2, caspase-1, and NLRP3, are also thought to play a significant role in the development of the inflammatory responses in ALI [35–37]. The formation of NLRP3 inflammasome can activate procaspase-1 to generate caspase-1, thereby promoting the maturation and secretion of inflammatory cytokine IL-1β [38]. Numerous serious human diseases, such as asthma, allergic airway inflammation, COVID-19 and gout are correlated with the overactivation of the NLRP3 inflammasome [39].

NO production inhibition assay was a useful bioassay for finding anti-inflammatory compounds and extracts [40]. In this study, it was found that the EtOAc fraction of the ethanol extract and the water extract of *T. chinense* had significant anti-inflammatory activity.

*T. chinense*, is frequently used by the local resident to treat a wide range of inflammatory disorders. Flavonoids, alkaloids, and organic acids are additionally regarded as the principal components [8]. But only a limited amounts of flavonoids, alkaloids and organic acids had been discovered in *T. chinense* [9, 10, 13]. Herein, our study discovered seven new compounds (**1**–**7**) and compounds **10**–**11**, **13**–**18**, **20**–**24**, **26**–**27**, **29**–**39**, **41**–**58**, **60**, and **62**–**63** were isolated from this plant for the first time, which enriched the chemical diversity of *T. chinense*.

In addition, there were few active compounds discovered in *T. chinense*, and limited evidence of pharmacological mechanism were reported. In the present study, we found twelve active compounds (**1**, **2**, **4**, **5**, **8–11**, **27**, **34**, **39** and **44**) from the EtOAc fraction and thirteen active compounds (**7**, **45**–**54**, **57** and **58**) from the water extract by using NO production inhibition assay. Flavonoids and fatty acids demonstrated strong inhibitory activity, while lignans, flavonoids, fatty acids, alkaloids, coumarins, phenylpropionic acid, and simple aromatics displayed moderate inhibitory activity. These findings preliminarily revealed the material basis of *T. chinense* against inflammatory diseases.

A network pharmacology analysis was constructed to further explore the potential targets of *T. chinense* against lung inflammation. We gathered constituents and their active targets to construct the network pharmacology analysis. However, in previous studies related to the *T. chinense*, only 34 compounds were identified [41]. Our research has firstly identified 54 compounds from *T. chinense*, and integrated them into the analysis, which significantly enriched the compositional data and made the analysis more authentic and reliable. KN (**19**), AG (**12**) and KF (**8**), respectively known as “Bai Rui Cao Su I” (百蕊草素I), “Bai Rui Cao Su II”(百蕊草素II), and “Bai Rui Cao Su III”(百蕊草素III) in Chinese, are the main constituents of *T. chinense* [42, 43]. KN (**19**) and AG (**12**) did not exhibit anti-inflammatory activity in RAW 264.7 cells induced by LPS, but demonstrated this activity in vivo. Additionally, in the network pharmacology analysis, they did not appear in the list of the top ten active compounds. Interestingly, their aglycone, kaempferol, exhibited strong activity both in vitro and in vivo, and it ranked as the third most active constituent in the network analysis (Fig. 4 and 11; Additional file 1: Table S3). We speculated that this might be due to changes in the physical properties of the flavonoid glycosides within the body. These changes could lead to a decrease in oral absorption, preventing them from crossing cell membranes. However, in the stomach, these flavonoid glycosides could be hydrolyzed at the glycosidic bond, thereby increasing the likelihood of their absorption.

Our study explored the anti-inflammatory activity of KN (**19**), AG (**12**) and KF (**8**) in ALI mice. The most numerous white blood cells in circulation are neutrophils and the site of inflammation will cause the aggregation of immune cells [44]. An overactive immune response can result in an imbalance between pro- and anti-inflammatory processes, which will then cause tissue damage [45]. Numerous cytotoxic substances, such as different pro-inflammatory cytokines, are produced by activated neutrophils, which will induce lung tissue damage [46]. In this study, we found that CE, BG, KN (**19**), AG (**12**) and KF (**8**) could reduce the number of leucocytes and neutrophils in ALI mice, which indicated that they could lessen the amount of alveolar neutrophil infiltration by preventing the production and recruitment of neutrophils. Furthermore, the level of IL-1β can significantly rise in the BALF of animals with ALI and overexpression of IL-1β tends to induce acute inflammation and alveolar tissue destruction [47]. The result showed that the CE, BG, and flavonoids of *T. chinense* might relieve LPS-induced ALI by preventing the production of IL-1β. KN (**19**) and KF (**8**) have anti-inflammatory activity by inhibiting the mitogen-activated protein kinase (MAPK) and nuclear transcription factors-κB (NF-κB) [48, 49]. AG (**12**) also attenuated the inflammatory response in the LPS-induced ALI mice model by decreasing not only the generation of TNF-α, IL-6, and IL-1β but also the activation of NF-κB [50]. In addition, inhibition of COX-2 reduces NLRP3 inflammasome-derived IL-1β secretion and macrophage pyrogenesis [51]. However, there is no evidence to suggest that the three compounds mentioned above can attenuate LPS-induced ALI through the NLRP3 inflammasome. According to our results, these three compounds together with CE and BG could alleviate the ALI by inhibiting NLRP3-related genes (e.g. NLRP3, caspase-1, IL-1β and COX-2).

The occurrence of adverse reactions or events associated with Traditional Chinese Medicine (TCM) not only poses a risk to the safe utilization of these medicines by the general public but also impedes the healthy growth of the TCM industry, its commercial ventures, and the process of internationalization [52]. Subacute toxicity, is often defined as functional or structural damage to an organism within a relatively short period or a brief exposure to a toxic agent (ranging from several days to a few months) [53]. Even drugs with relatively low toxicity may accumulate within the body over time due to environmental persistence or long-term human consumption, leading to potential adverse health effects and toxic reactions that cannot be dismissed. *T. chinense*, was subjected to an extensive literature review that failed to reveal systematic evidence of toxicity [41]. BG serves as a prime example of a natural herb successfully transformed into a widely used clinical product. Nonetheless, clinical trial records for BG have reported several adverse reactions linked to the gastrointestinal tract. Besides, evaluating the safety of the BG contributes to validating regulatory approval, reconciling discrepancies between nonclinical and clinical data, elucidating mechanisms of adverse effects, and providing assurance for human multiple dosing or large-scale clinical trials [54]. Thus, we conducted a 28-day subacute toxicity experiment involving CE and BG.

The BG clinical dosage used was 5 g/3 times/day, a selection made while considering granule solubility and applying Young's rule (child age/(child age + 12) × adult dose) for dose conversion in adults and children (assuming an adult weighing 70 kg and a child aged 8 years) [55]. Consequently, we selected doses of 4.875 g/kg and 9.75 g/kg in the BG group, which equate to one and twofold of clinically administered dose, respectively. For the CE group, we selected a dosage of 60 g, following the recommendations in ‘*Zhonghua Bencao*’ [56] for the treatment of chronic bronchitis. Therefore, we set the dosage for the CE at 11.14 g/kg, which is equivalent to fourfold clinical dosage, taking into account the 23.8% yield of the CE and the specifications of the granules.

Changes in body weight and food intake observed during the toxicity study were closely monitored as comprehensive indicators of the test substances impact on the animal pathophysiological condition [57]. In comparison to the control group, the four treatment groups exhibited no notable abnormalities, alterations in behavior, variations in body weight, shifts in food and water consumption, or instances of mortality.

Toxicity testing relies on standard blood markers as essential tools for assessing the impact of toxic substances on the hematological system [53]. These toxicants or pharmaceutical agents have the potential to induce conditions such as leukopenia (reduced white blood cell count), anemia (reduced red blood cell count, including hemoglobin and hematocrit levels), immunosuppression (decreased lymphocyte count), or hemorrhagic tendencies (irregularities in platelet-related parameters). Notably, our analysis revealed that none of the experimental groups exhibited statistically significant deviations in standard blood parameters. Furthermore, comprehensive data acquired from serum biochemical analyses, aimed at quantifying levels of TBA, ALP, ALT, AST (well-recognized markers for liver and biliary health), BUN and CREA (markers of kidney function), CK and LDH (commonly employed biomarkers for assessing cardiac and muscular health among other functions) similarly exhibited no statistically significant disparities.

It is worth noting that toxic substances typically distribute to specific organs upon entering an animal's body, and certain organs may become primary target sites for toxic effects, potentially resulting in variations in organ coefficients [58]. Organ coefficients provide valuable insights into the cumulative toxic impacts of chemical agents on respective organs and serve as critical clues in identifying the primary target organ of toxicity [59]. The organ coefficients of the major organs in the treatment group showed no significant variations compared to those in the normal control group.

In light of the gastrointestinal adverse effects mentioned in the clinical instructions, we conducted histological analyses of vital organs, including the heart, liver, spleen, lungs, stomach, and intestines, using H&E-stained sections. Our findings revealed no statistically significant differences between the treatment group and the control group. This evidence, combined with the organ coefficient data, strongly suggested that extracts, granules, derived from *T. chinense* have minimal impact on organ tissues.

In summary, a total of sixty-three compounds were discovered from *T. chinense*, including seven new compounds and forty-seven compounds were isolated from *T. chinense* for the first time. Among them, the new compounds **1**, **2**, **4** and **5** as well as a variety of other compounds had NO production inhibition activity, including flavonoids, fatty acids lignans, alkaloids, coumarins, phenylpropionic acids, and simple aromatics. In addition, both flavonoid KF (**8**) and its glycosides KN (**19**) and AG (**12**) from *T. chinense* could alleviate lung inflammation caused by LPS-induced ALI in mice, which might be related to the regulation of NLRP3, caspase-1, IL-1β, and COX-2. No toxicity of *T. chinense* and Bairui Granules was observed under the twofold of clinically used doses. Collectively, our research discovered the active constituents and verified the safety of *T. chinense* and Bairui Granules that support their clinical and traditional applications. Above results not only provided scientific basis for the clinical treatment of inflammatory diseases with *T. chinense* and its preparations, but also verified the potential of its representative flavonoid glycosides to be further developed into drugs for treating pulmonary inflammatory diseases.

### Supplementary Information

**Additional file 1.** Additional figures and tables.

## Data Availability

The datasets used and/or analyzed during the current study are available from the corresponding author on reasonable request.

## Abbreviations

AG: Astragalin
ALT: Alanine aminotransferase
ALI: Acute lung inflammation
ARDS: Acute respiratory distress syndrome
AST: Aspartate aminotransferase
ALP: Alkaline phosphatase
BG: Bairui Granules
BALF: Bronchoalveolar Lavage Fluid
BUN: Blood urea nitrogen
CE: Crude ethanol extract
COPD: Chronic obstructive pulmonary disease
COX-2: Cyclooxygenase-2
CK: Creatine kinase
CREA: Creatinine
DCM: Dichloromethane
DMSO: Dimethyl sulfoxide
DIDOX: 3,4-Dihydroxy-benzohydroxamic acid
DEX: Dexamethasone
EtOAc: Ethyl acetate
Gene ontology: GO
H&E: Hematoxylin and eosin
IL-1β: Interleukin-1β
KN: Kaempferol-3-*O*-glucorhamnoside
KF: Kaempferol
LPS: Lipopolysaccharide
LDH: Lactate dehydrogenase
MIR: Maximum inhibition rate
MPV: Mean platelet volume
MCH: Mean corpuscular hemoglobin
NLRP3: NOD-like receptor protein 3
Neut/gran: Neutrophil-to-granulocyte ratio
NO: Nitric oxide
NF-κB: Nuclear transcription factors-κB
PE: Petroleum ether
PPI: Protein–protein interaction
qRT-PCR: Quantitative Real-Time Polymerase Chain Reaction
RBC: Red blood cell count
SARS-CoV-2: Syndrome-coronavirus-2
TBA: Total bile acids
WBC: White blood cell count

## References

[1] Fan E, Brodie D, Slutsky AS (2018). Acute respiratory distress syndrome advances in diagnosis and treatment. JAMA-J Am Med Assoc.

[2] Phua J, Badia JR, Adhikari NK (2009). Has mortality from acute respiratory distress syndrome decreased over time?: A systematic review. Am J Respir Crit Care Med.

[3] Grommes J, Soehnlein O (2011). Contribution of neutrophils to acute lung injury. Mol Med.

[4] Butt Y, Kurdowska A, Allen TC (2016). Acute lung injury a clinical and molecular review. Arch Pathol Lab Med.

[5] Huang CL, Wang YM, Li XW (2020). Clinical features of patients infected with 2019 novel coronavirus in Wuhan. China Lancet.

[6] Zhang J (1987). Microscopic Identification of *Thesium chinense*. J Chin Med Mater..

[7] Editorial Board of Chinese Materia Medica (1999). Chinese Materia Medica.

[8] Bairuicao Research Group. Chemical constituents of *Thesium chinense* Turcz. Zhongcaoyao Tongxun. 1976:342–48.

[9] Liu C, Li XT, Cheng RR (2018). Anti-oral common pathogenic bacterial active acetylenic acids from *Thesium chinense* Turcz. J Nat Med.

[10] Lee IK, Kim KH, Choi SU (2009). Phytochemical constituents of *Thesium chinense* TURCZ and their cytotoxic activities *in vitro*. Nat Prod Sci.

[11] Liu Y, Zahida P, Deng Y-L (2009). Study on the flavonoids compounds of *Thesium chinese*. J Chin Med Mater.

[12] Zou Y, Hong M, Yang X (2016). Isolation of Chemical Components from *Thesium chinense*. Chin J Exp Tradit Med Formulae.

[13] Wang Z, Li S (2006). Isolation and identification of alkaloids from *Thesium chinense* Turcz. Chin J Med Chem.

[14] Lu Y, Wang S (2004). Study on chemical constituents of *Thesium chinensis*. Chin Trad Herbal Drugs.

[15] Liu ZZ, Ma JC, Deng P (2023). Chemical constituents of *Thesium chinense* Turcz and their *in vitro* antioxidant, anti-Inflammatory and cytotoxic activities. Molecules.

[16] Yuan Y, Long Z, Xu X (2006). Comparison of wild and cultured *Thesium Chinense* Turcz on bacteriostasis and anti-inflammation. Pharm Biotechnol..

[17] Parveen Z, Deng Y, Saeed MK, et al. Antiinflammatory and analgesic activities of *Thesium chinense* Turcz. extracts and its major flavonoids, kaempferol and kaempferol-3-*O*-glucoside. Yakugaku Zasshi. 2007;127(8):1275–79. 10.1248/yakushi.127.1275 17666881

[18] Wang J, Wang Y, Liu H (2018). Clinical effect observation of Bairui Granules combined with amoxicillin and clavulanate potassium tablets in treating acute tonsillitis. Chin Tradi Herbal Drugs.

[19] Feng Y, Fu C, Fu M (2020). Clinical study of Bairui Granules combined with Cefaclor Granules in treatment of acute bronchitis in children. Drug Eval Res.

[20] Zhou YK, Yang JQ, Zhao RP (2023). Observational study of Bairui Granules as adjunctive treatment for severe pneumonia in 150 children. J Ped Trad Chin Med.

[21] Deng LP, Yang Y, Wang J (2022). Effect of Bairui Granules on lung function and serum levels of COX-2 and sTREM-1 in patients with chronic obstructive pulmonary disease. World J Integr Trad West Med..

[22] National Administration of Traditional Chinese Medicine of China (2012). Guidelines for diagnosis and treatment of common diseases of otorhinolaryngology in traditional Chinese medicine.

[23] Wang Y, Chao E, Wang G (2015). Guidelines for Clinical Application of Chinese Medicines-Infectious Diseases.

[24] Kim CS, Bae M, Oh J (2017). Anti-neurodegenerative biflavonoid glycosides from *Impatiens balsamina*. J Nat Prod.

[25] Sun XZ, Zimmermann ML, Campagne JM (2000). New sucrose phenylpropanoid esters from *Polygonum perfoliatum*. J Nat Prod.

[26] Sun DW, Cao F, Liu M (2017). New fatty acid From a gorgonian-derived Xylaria sp. fungus. Chem Nat Compd..

[27] Bourjot M, Leyssen P, Eydoux C (2012). Chemical constituents of *Anacolosa pervilleana* and their antiviral activities. Fitoterapia.

[28] Ibragimov BT, Talipov SA, Tishchenko GN (1981). Molecular and crystal structure of tetrahydroneosophoramine. Chem Nat Compd.

[29] Shao BZ, Xu ZQ, Han BZ (2015). NLRP3 inflammasome and its inhibitors: a review. Front Pharmacol.

[30] Cui JH, Jia JP (2021). Natural COX-2 inhibitors as promising anti-inflammatory agents: an update. Curr Med Chem.

[31] Long ME, Mallampalli RK, Horowitz JC (2022). Pathogenesis of pneumonia and acute lung injury. Clin Sci.

[32] Ding YH, Song YD, Wu YX (2019). Isoalantolactone suppresses LPS-induced inflammation by inhibiting TRAF6 ubiquitination and alleviates acute lung injury. Acta Pharmacol Sin.

[33] Wang FZ, Kream RM, Stefano GB (2020). Long-term respiratory and neurological sequelae of COVID-19. Med Sci Monit.

[34] Mokra D (2020). Acute lung injury-from pathophysiology to treatment. Physiol Res.

[35] Liu YH, Shang LR, Zhou JB (2022). Emodin attenuates LPS-induced acute lung injury by inhibiting NLRP3 inflammasome-dependent pyroptosis signaling pathway *in vitro* and *in vivo*. Inflammation.

[36] Ying YG, Mao Y, Yao M (2019). NLRP3 inflammasome activation by microRNA-495 promoter methylation may contribute to the progression of acute lung injury. Mol Ther-Nucl Acids.

[37] Al-Harbi NO, Imam F, Al-Harbi MM (2016). Dexamethasone attenuates LPS-induced acute lung injury through inhibition of NF-κB, COX-2, and pro-inflammatory mediators. Immunol Invest.

[38] Swanson KV, Deng M, Ting JPY (2019). The NLRP3 inflammasome: molecular activation and regulation to therapeutics. Nat Rev Immunol.

[39] Mangan MSJ, Olhava EJ, Roush WR (2018). Targeting the NLRP3 inflammasome in inflammatory diseases. Nat Rev Drug Discov.

[40] Zhu SS, Zhang YF, Ding M (2022). Anti-neuroinflammatory components from *Clausena lenis* Drake. Molecules.

[41] Li GH, Fang KL, Yang K (2021). Thesium chinense Turcz.: An ethnomedical, phytochemical and pharmacological review. J Ethnopharmacol..

[42] Chu XQ, Hu YQ, Yue L (2013). Purification of total flavonoids from the extracts of Thesium chinese Turcz with macroporous adsorption resin. J Chin Med Mater..

[43] Yz Z (2018). Comparative study on the quality of Bairui Pills from different manufacturers. Beijing J Tradit Chin Med.

[44] Amulic B, Cazalet C, Hayes GL et al. Neutrophil function: from mechanisms to disease. In: Annu Rev Immunol*.* Edited by Paul WE, vol. 30. Palo Alto: Annual Reviews; 2012: 459–89. 10.1146/annurev-immunol-020711-074942 22224774

[45] Qian YS, Wang ZW, Lin HR (2022). TRIM47 is a novel endothelial activation factor that aggravates lipopolysaccharide-induced acute lung injury in mice via K63-linked ubiquitination of TRAF2. Signal Transduct Target Ther.

[46] Yang SC, Tsai YF, Pan YL (2021). Understanding the role of neutrophils in acute respiratory distress syndrome. Biomed J.

[47] Ju MJ, Liu BF, He HY (2018). MicroRNA-27a alleviates LPS-induced acute lung injury in mice via inhibiting infLammation and apoptosis through modulating TLR4/MyD88/NF-κB pathway. Cell Cycle.

[48] Sun ZJ, Li Q, Hou RR (2019). Kaempferol-3-*O*-glucorhamnoside inhibits inflammatory responses via MAPK and NF-κB pathways *in vitro* and *in vivo*. Toxicol Appl Pharmacol.

[49] Chen XJ, Yang XF, Liu TJ (2012). Kaempferol regulates MAPKs and NF-κB signaling pathways to attenuate LPS-induced acute lung injury in mice. Int Immunopharmacol.

[50] Soromou LW, Chen N, Jiang LN (2012). Astragalin attenuates lipopolysaccharide-induced inflammatory responses by down-regulating NF-κB signaling pathway. Biochem Biophys Res Commun.

[51] Hua KF, Chou JC, Ka SM (2015). Cyclooxygenase-2 regulates NLRP3 inflammasome-derived IL-1β production. J Cell Physiol.

[52] Bai Z-F, Wang J-B, Xiao X-H (2022). Cognition innovation of toxicity of Chinese medicine and safe and precise medication. China J Chin Materia Med.

[53] Fritsche E, Haarmann-Stemmann T, Kapr J (2021). Stem cells for next level toxicity testing in the 21st century. Small.

[54] Bass AS, Hombo T, Kasai C et al. A historical view and vision into the future of the field of safety pharmacology. In: Principles of Safety Pharmacology*.* Edited by Pugsley MK, Curtis MJ, vol. 229: Springer-Verlag Berlin, Heidelberger Platz 3, D-14197 Berlin, Germany; 2015: 3–45. 10.1007/978-3-662-46943-9_1 26091634

[55] Wade CI, Martinez T. Young's Rule. In: StatPearls*.* Treasure Island (FL): StatPearls Publishing; 2023. 32119490

[56] State Administration of Traditional Chinese Medicine (1999). Zhong Hua Ben Cao.

[57] Atmane SA, Eldjoudi DA, Ozbek ZA (2022). Acute and 28-day repeated dose toxicity evaluations of cold pressed Pinus halepensis Mill seed oil in mice and rats. Regul Toxicol Pharm..

[58] Wu P, Lin S, Cao G (2022). Absorption, distribution, metabolism, excretion and toxicity of microplastics in the human body and health implications. J Hazard Mater.

[59] Lazic SE, Semenova E, Williams DP (2020). Determining organ weight toxicity with Bayesian causal models: Improving on the analysis of relative organ weights. Sci Rep.

