# Supplementary material for: Systematic investigation of the material basis, effectiveness and safety of Thesium chinense Turcz. and its preparation Bairui Granules against lung inflammation
Source: Chin Med. 2024 May 8;19:67. doi: 10.1186/s13020-024-00940-y (PMC11080236; doi:10.1186/s13020-024-00940-y)
Supplement: Supplementary file 1 — Additional file 1. Additional figures and tables. [file 13020_2024_940_MOESM1_ESM.docx]

**Additional data**

**Systematic investigation of the material basis,** **effectiveness and safety of *Thesium chinense* Turcz. and its preparation Bairui Granules against lung inflammation**

Guang-Cheng Peng^1^, Jin-Hua Hao^1^, Yue-Qin Guan^2^, Ying-Yue Wang^1^, Ming-Jie Liu^1^, Guo-Hui Li^1,3**^, Zhen-Peng Xu^1^, Xue-Sen Wen^1^, Tao Shen^1,*^

**Affiliation**

1 Key Lab of Chemical Biology (MOE), School of Pharmaceutical Sciences, Cheeloo College of Medicine, Shandong University, Jinan, People’s Republic of China

2 Jiuhua Huayuan Pharmaceutical Co., Ltd., Chuzhou, People’s Republic of China

3 Department of Pharmacy, Jinan Maternity and Child Care Hospital Affiliated to Shandong First Medical University, Jinan, People’s Republic of China

**Correspondence**

* Key Lab of Chemical Biology (MOE), School of Pharmaceutical Sciences, Shandong University, 44 Wenhua Xi Road, Jinan 250012, People’s Republic of China.

** Jinan Maternity and Child Care Hospital Affiliated to Shandong First Medical University, Jinan, People’s Republic of China.

E-mail address: v_liguohui@126.com (G.-H. Li), shentao@sdu.edu.cn (T. Shen); Phone: 86-531-88382028; Fax: 86-531-88382548.

**Contents**

**1. Detailed isolation process**

**2. Inhibitory activity of crude extracts on LPS-induced NO production in RAW 264.7 cells**

**3. Spectra for the isolated new compounds**

**4. Data for serum biochemical indices and blood routines**

**5. Data for RT-PCR**

**6.** **Comparison between known compounds and reported literature**

**7. Data for network pharmacology analysis**

**8. References**

**1. Detailed isolation process**

E3 (4.4 g) was separated by Middle Chromatogram Isolated (MCI) gel eluted with a gradient solvent system of MeOH−H_2_O (3:7 → 1:0) to yield eight subfractions (E3a-h) and compound **8** (750.0 mg) was obtained from E3e (0.8 g) by crystalizing. E3a (0.2 g) was subjected to Sephadex LH-20 column (MeOH) followed by crystalizing to obtain **39** (36.9 mg). The crystallization mother liquor was further purified by silica gel column eluted with PE−EtOAc−acetic acid (10:8:0.1) followed by semipreparative HPLC (MeOH−H_2_O−formic acid, 85:15:0.1) to afford **44** (4.9 mg, t_R_ = 30 min). E3b (0.6 g) was subjected to Sephadex LH-20 column (MeOH) to obtain four subfractions (E3b1-4). E3b1 (0.2 g) was purified by semipreparative HPLC (MeOH−H_2_O−formic acid, 30:70:0.1) to obtain **4** (2.9 mg, t_R_ = 49 min). E3b3 (33.5 mg) was purified by semipreparative HPLC (MeOH−H_2_O−formic acid, 16:84:0.1) to obtain **42** (2.6 mg, t_R_ = 51 min) and **37** (6.8 mg, t_R_ = 55 min). E3b4 (68.7 mg) was purified by semipreparative HPLC (MeOH−H_2_O−formic acid, 21:79:0.1) to obtain **40** (15.7 mg, t_R_ = 39 min), **41** (10.8 mg, t_R_ = 52 min) and **26** (8.5 mg, t_R_ = 56 min). Compound **28** (482.8 mg) was obtained from the subfractions E3c (0.3 g), which was purified by Sephadex LH-20 column (MeOH). E3d (0.3 g) was chromatographed on Sephadex LH-20 column (MeOH) followed by semipreparative HPLC (MeOH−H_2_O−formic acid, 35:65:0.1) to yield **27** (35.0 mg, t_R_ = 40 min). E3f (0.1 g) was applied to Sephadex LH-20 column (MeOH) followed by semipreparative HPLC (MeOH−H_2_O−formic acid, 70:30:0.1) to afford **5** (4.8 mg, t_R_ = 40 min).

E4 (1.8 g) also had anti-inflammatory activity and was subjected to MCI gel eluted with MeOH−H_2_O (3:7 → 1:0) to give nine subfractions (E4a-i). E4b (97.1 mg) was loaded onto a Sephadex LH-20 column (MeOH) to obtain **43** (5.2 mg) and the subfraction E4b2 (40.5 mg), which was further purified by semipreparative HPLC (MeOH−H_2_O−formic acid, 10:90:0.1) to afford **35** (2.5 mg, t_R_ = 9 min). E4c (0.3 g) was separated by Sephadex LH-20 column (MeOH) to give five subfractions (E4c1-5). E4c3 (41.2 mg) was purified by semipreparative HPLC (MeOH−H_2_O−formic acid, 27:73:0.1) to yield **36** (2.4 mg, t_R_ = 36 min) and E4c5 (44.8 mg) was purified by semipreparative HPLC (MeOH−H_2_O−formic acid, 20:80:0.1) to yield **25** (4.7 mg, t_R_ = 51 min). E4d (0.3 g) was chromatographed on Sephadex LH-20 column (MeOH) followed by semipreparative HPLC (MeOH−H_2_O−formic acid, 30:70:0.1) to yield **32** (11.7 mg, t_R_ = 62 min). E4e (0.2 g) was applied to a Sephadex LH-20 column (MeOH) to obtain **9** (9.7 mg) and the subfraction E4e5 (14.2 mg), which was further purified by semipreparative HPLC (MeOH−H_2_O−formic acid, 55:45:0.1) to afford **10** (11.7 mg). E4f (0.17 g) were subjected to Sephadex LH-20 column (MeOH) followed by crystalizing to obtain **11** (2.8 mg).

E5 (2.1 g) was applied to the MCI gel column MeOH−H_2_O (3:7 → 1:0) to offer eight subfractions (E5a-h). E5d (0.2 g) was chromatographed on Sephadex LH-20 column (MeOH) followed by semipreparative HPLC (MeOH−H_2_O−formic acid, 45:55:0.1) to yield **16** (4.8 mg, t_R_ = 46 min). E5e (0.7 g) was subjected to silica gel column eluted with a gradient solvent system of DCM−MeOH (30:1 → 2:1) and next purified by semipreparative HPLC (MeOH−H_2_O−formic acid, 51:49:0.1) to obtain **17** (7.7 mg, t_R_ = 38 min). E5f (0.4 g) was loaded onto a Sephadex LH-20 column (MeOH) followed by semipreparative HPLC (MeOH−H_2_O−formic acid, 51:49:0.1) to afford **18** (3.1 mg, t_R_ = 22 min), **30** (5.5 mg, t_R_ = 45 min).

E6 (2.4 g) was separated by MCI gel column MeOH−H_2_O (3:7 → 1:0) to obtain eleven subfractions (E6a-k). E6a (78.6 mg) was subjected to Sephadex LH-20 column (MeOH) and further purified by precipitating to yield **38** (1.8 mg). Compound **12** (919.6 mg) was obtained from E6f (1.2 g) by crystallizing and the subfraction E6f7 (0.1 g) was purified by silica gel column eluted with DCM−MeOH (100:1 → 0:1) and Sephadex LH-20 column (MeOH) to yield **22** (34.4 mg). E6g (0.1 g) was chromatographed on silica gel column eluted with DCM−MeOH (30:1 → 0:1) followed by Sephadex LH-20 column (MeOH) to afford four subfractions (E6g3a-d). E6g3b (6.0 mg) was applied to semipreparative HPLC (MeOH−H_2_O, 55:45) to obtain **24** (2.6 mg, t_R_ = 22 min) and E6g3d (22.5 mg) was subjected to semipreparative HPLC (MeOH−H_2_O, 49:51) to yield **15** (19.1 mg, t_R_ = 29 min). E6h (0.2 g) was subjected to silica gel column eluted with DCM−MeOH (100:1 → 0:1) followed by Sephadex LH-20 column (MeOH) and then semipreparative HPLC (MeOH−H_2_O, 55:45) to afford **14** (10.1 mg, t_R_ = 27 min).

E7 (12.0 g) was loaded onto an MCI gel column MeOH−H_2_O (3:7 → 1:0) to offer eight subfractions (E7a-h). E7a (0.5 g) was subjected to silica gel column eluted with DCM−MeOH (50:1 → 0:1) followed by Sephadex LH-20 column (MeOH) to obtain **33** (13.3 mg) and the subfraction E7a5e (37.3 mg), which was further purified by silica gel column eluted with DCM−MeOH−formic acid (10:1:0.1) to afford **34** (13.0 mg). E7b (1.1 g) was chromatographed on silica gel column eluted with DCM−MeOH (50:1 → 0:1) to obtain six subfractions (E7b1-6) and **19** (636.2 mg) was precipitated in E7b5 (0.7 g). E7b3 (0.1 g) was applied to Sephadex LH-20 column (MeOH) and then purified by semipreparative HPLC (MeOH−H_2_O−formic acid, 25:75:0.1) to afford **29** (7.4 mg, t_R_ = 27 min**)**, **31** (5.0 mg, t_R_ = 47 min**)**. E7c (5.5 g) was subjected to silica gel column eluted with DCM−MeOH (20:1 → 0:1) to give five subfractions (E7c1-5). E7c2 (1.3 g) was chromatographed on Sephadex LH-20 column (MeOH) to afford nine subfractions (E7c2a-i). Compound **23** (73.9 mg, t_R_ = 45 min) was purified by semipreparative HPLC (MeOH−H_2_O, 40:60) from E7c2b (0.5 g) and Compound **21** (25.8 mg, t_R_ = 36 min) was purified by semipreparative HPLC (MeOH−H_2_O−formic acid, 42:58:0.1) from E7c2d (0.1 g). E7c3 (1.4 g) was loaded onto an Sephadex LH-20 column (MeOH) to obtain **13** (23.1 mg) and the subfraction E7c3d (0.9 g) was further purified by semipreparative HPLC (MeOH−H_2_O, 45:55) to obtain **20** (34.2 mg, t_R_ = 42 min). E7d (0.8 g) was subjected to silica gel column eluted with DCM−MeOH (30:1 → 0:1) to offer six subfractions (E7d1-6). E7d3 (0.2 g) was chromatographed on Sephadex LH-20 column (MeOH) and then purified by semipreparative HPLC (MeOH−H_2_O−formic acid, 45:55:0.1) to afford **3** (37.8 mg, t_R_ = 47 min). E7d5 (0.1 g) was purified by Sephadex LH-20 column (MeOH) and semipreparative HPLC (MeOH−H_2_O−formic acid, 37:63:0.1) to obtain **1** (2.5 mg, t_R_ = 34 min) and **2** (2.1 mg, t_R_ = 36 min).

S5 (0.2 g) was loaded onto a C_18_ column eluted with MeOH−H_2_O (0:1 → 1:0) to obtain six subfractions (S5a-f) and S5c (26.0 mg) was purified by semipreparative HPLC (MeOH−H_2_O, 45:55) to afford **45** (2.8 mg, t_R_ = 38 min), **58** (8.4 mg, t_R_ = 47 min), **59** (1.3 mg, t_R_ = 58 min). S5f (72.0 mg) was chromatographed on Sephadex LH-20 column (MeOH) and isolated by semipreparative HPLC with MeOH (10%) containing triethylamine phosphate buffer (PH = 3) to obtain **6** (27.2 mg, t_R_ = 18 min), **7** (13.0 mg, t_R_ = 22 min).

S6 (0.3 g) was subjected to C_18_ column eluted with MeOH−H_2_O (2:8 → 1:0) to obtain eight subfractions (S6a-h). S6c (63.4 mg) was subjected to Sephadex LH-20 column (MeOH) to afford three subfractions (S6c1-3) and then S6c1 (41.0 mg) was applied to semipreparative HPLC (CH_3_CN−H_2_O−formic acid, 17:83:0.1) to yield **60** (3.6 mg, t_R_ = 46 min). S6c2 (10.3 mg) was subjected to semipreparative HPLC (MeOH−H_2_O−formic acid, 30:70:0.1) to obtain **62** (1.4 mg, t_R_ = 42 min) and **63** (2.5 mg, t_R_ = 48 min). S6d (66.8 mg) was subjected to Sephadex LH-20 column (MeOH) followed by semipreparative HPLC (MeOH−H_2_O−formic acid, 44:56:0.1) to afford **48** (13.0 mg, t_R_ = 49 min) and **49** (5.2 mg, t_R_ = 49 min).

S7 (1.5 g) was chromatographed on silica gel column to afford nine subfractions (S7a-i). Then, S7b (45.0 mg) was purified by Sephadex LH-20 column followed by semipreparative HPLC (MeOH−H_2_O, 27:73) to obtain **55** (3.0 mg, t_R_ = 61 min) and **61** (1.1 mg, t_R_ = 40 min). S7c (0.3 g) was subjected to Sephadex LH-20 column (MeOH) followed by semipreparative HPLC (MeOH−H_2_O, 44:56) to obtain nine subfractions (S7c2a-i) and S7c2a (15.3 mg) was further purified by semipreparative HPLC (MeOH−H_2_O, 25:75) to yield **56** (3.7 mg, t_R_ = 84 min) and **57** (8.7 mg, t_R_ = 95 min). S7c2b (28.2 mg) and S7c2f (27.2 mg) was purified by crystallizing to yield **46** (15.6 mg) and **47** (27.0 mg). S7d (0.7 g) was subjected to Sephadex LH-20 column (MeOH) to obtain five subfractions (S7d1-5). S7d1 (0.3 g) was chromatographed on C_18_ column eluted with MeOH−H_2_O (2:8 → 1:0) followed by semipreparative HPLC (CH_3_CN−H_2_O−formic acid, 23:77:0.1) to obtain **54** (2.2 mg, t_R_ = 42 min). S7d2 (0.3 g) was purified by semipreparative HPLC (MeOH−H_2_O, 40:60) to yield **53** (75.1 mg, t_R_ = 52 min) and the subfraction S7d2b (109.6 mg) was further purified by semipreparative HPLC (MeOH−H_2_O−formic acid, 30:70:0.1) to yield **50** (8.8 mg, t_R_ = 56 min) and **51** (48.5 mg, t_R_ = 60 min). S7e (0.3 g) was chromatographed on Sephadex LH-20 column (MeOH) followed by semipreparative HPLC (MeOH−H_2_O, 30:70) to obtain **52** (14.1 mg, t_R_ = 44 min).

**2. Inhibitory activity of crude extracts on LPS-induced NO production in RAW 264.7 cells**

**
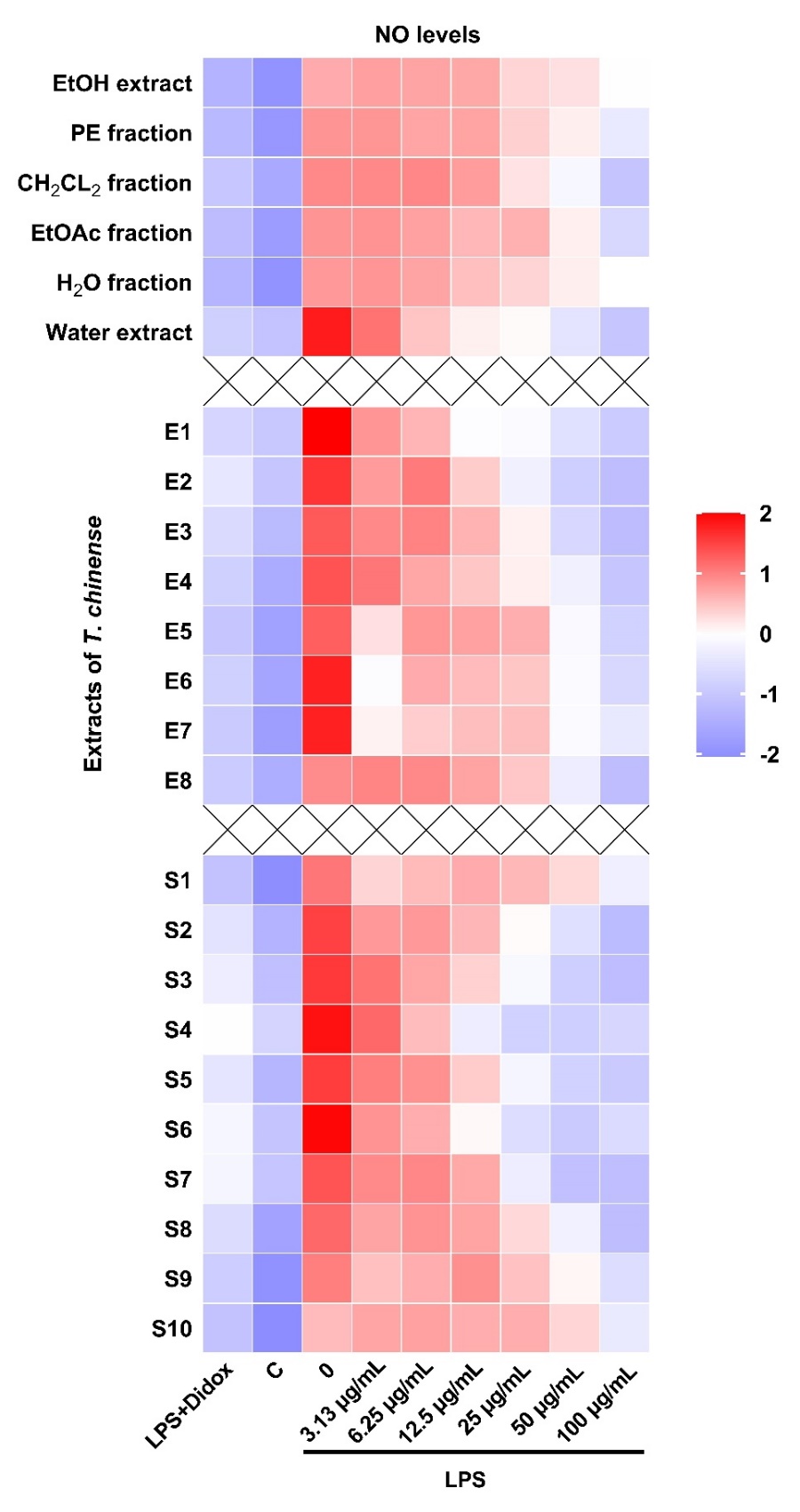
**

**Fig. S1.** Heat map analysis of inhibitory activity of crude extracts on LPS-induced NO production in RAW 264.7 cells. NO level was measured after treatment with compounds at indicated doses along with LPS (1 μg/mL) for 24 h. The data were normalized using Z-score (<https://www.omicshare.com/tools>). The color of the spots represents the level of NO production in each group, with red indicating a higher level and blue indicating a lower level. C: control group.

**3. Spectra for the isolated new compounds**


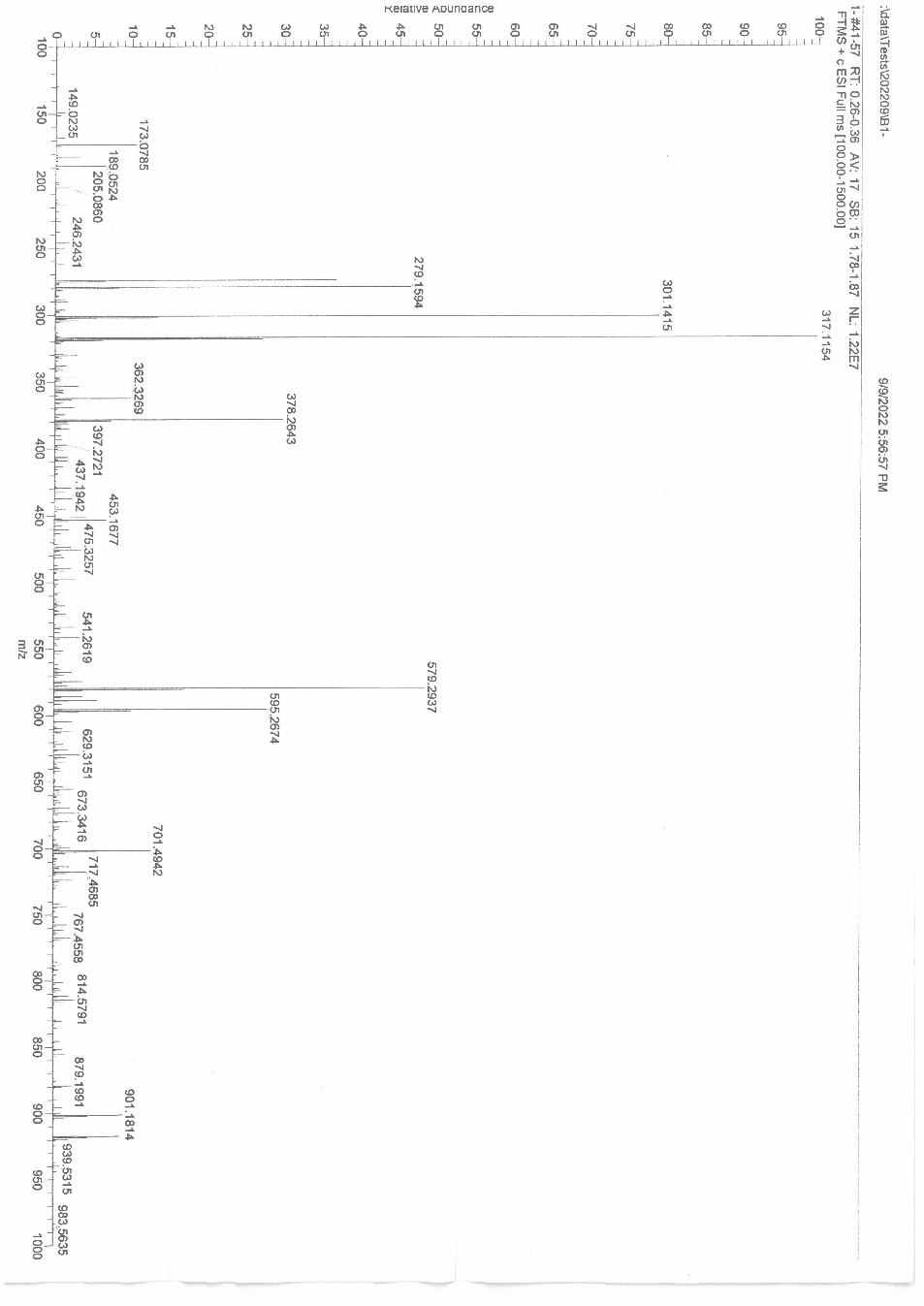


**Figure S2. HR-ESI-MS spectrum of 1.**

**Figure S3.** **^1^H NMR spectrum of 1 (600 MHz, CD_3_OD).**

**Figure S4. ^13^C NMR spectrum of 1 (150 MHz, CD3OD).**

**Figure S5. HSQC spectrum of 1.**

**Figure S6. ^1^H-^1^H COSY spectrum of 1.**

**Figure S7. HMBC spectrum of 1.**


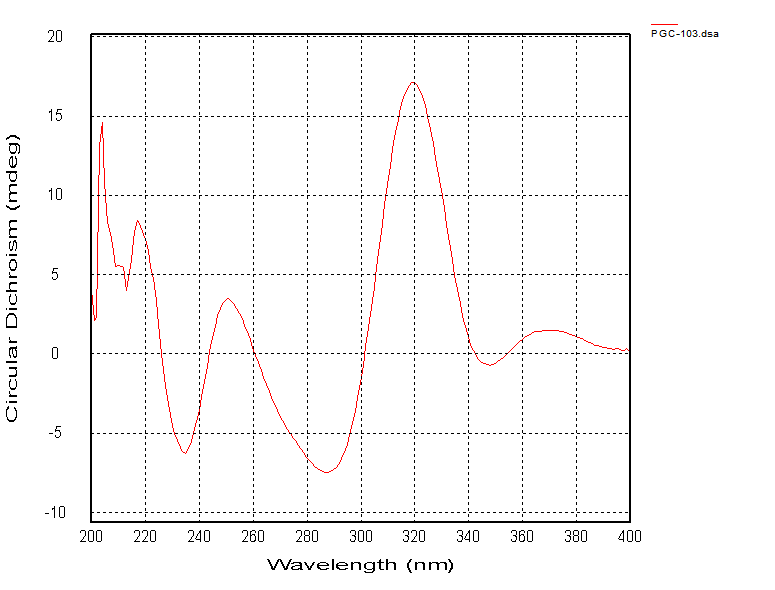


**Figure S8. ECD spectrum of 1.**

**
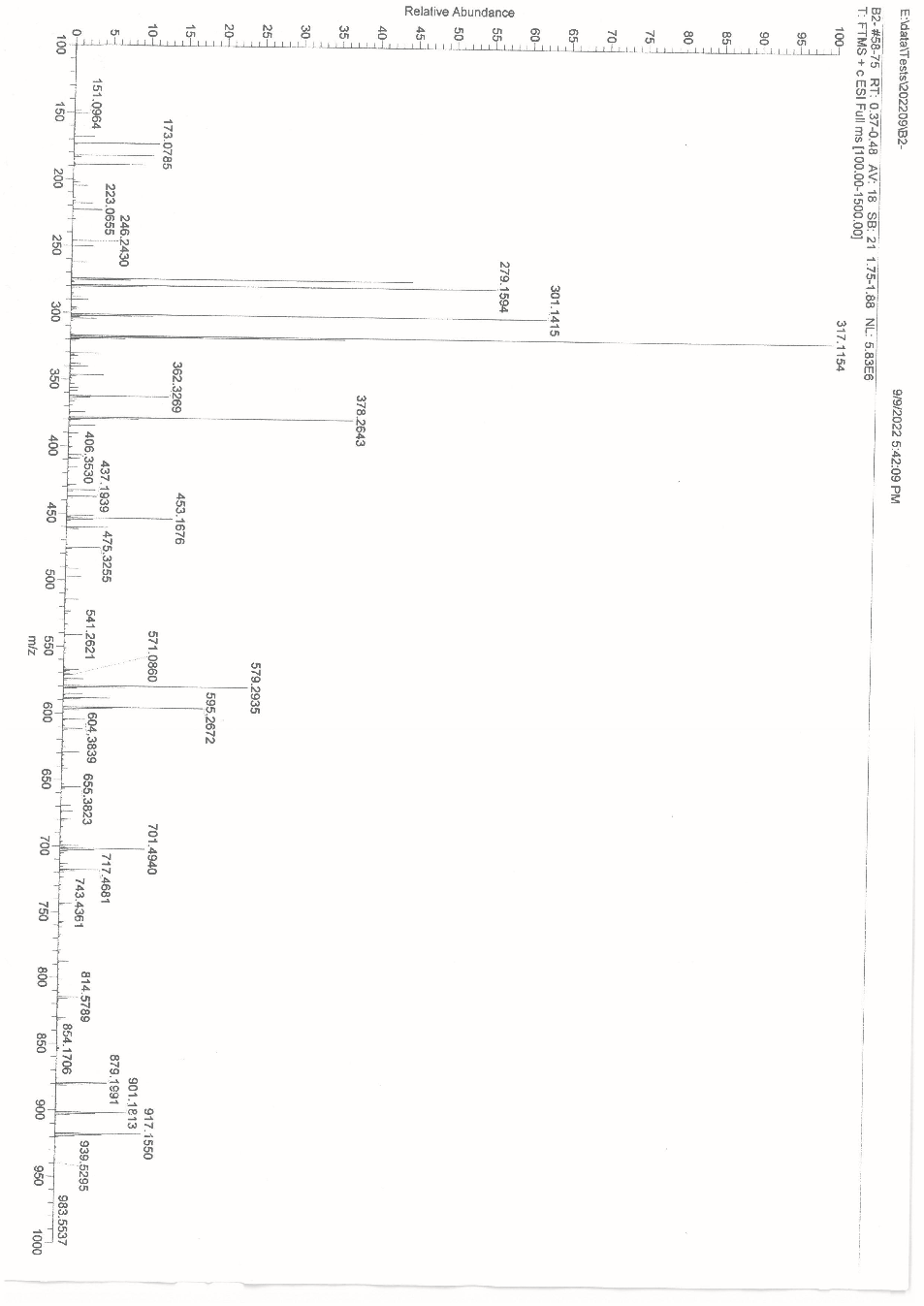
**

**Figure S9. HR-ESI-MS spectrum of 2.**

**Figure S10.** **^1^H NMR spectrum of 2 (600 MHz, CD_3_OD).**

**Figure S11. ^13^C NMR spectrum of 2 (150 MHz, CD_3_OD).**

**Figure S12. HSQC spectrum of 2.**

**Figure S13. ^1^H-^1^H COSY spectrum of 2.**

**Figure S14. HMBC spectrum of 2.**


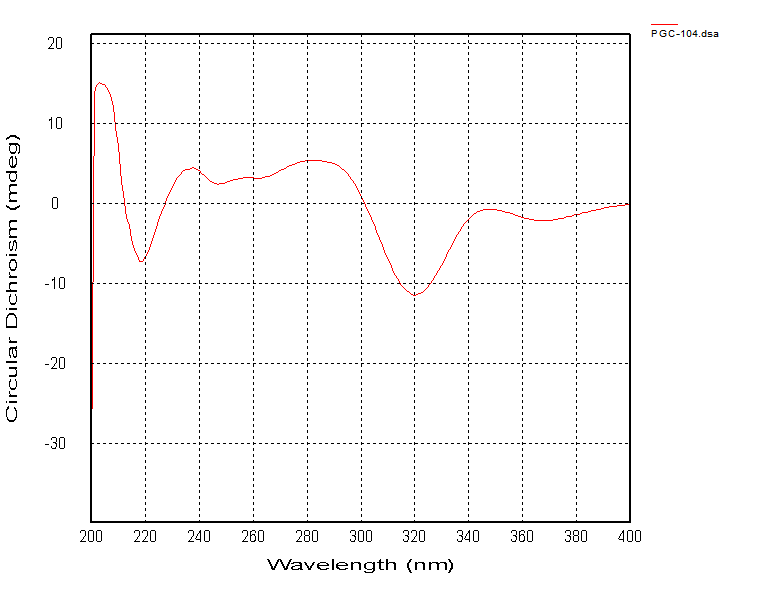


**Figure S15. ECD spectrum of 2.**

**Figure S16. HR-ESI-MS spectrum of 3.**

**Figure S17.** **^1^H NMR spectrum of 3 (600 MHz, CD_3_OD).**

**Figure S18. ^13^C NMR spectrum of 3 (150 MHz, CD_3_OD).**

**Figure S19. DEPT 135 spectrum of 3.**

**Figure S20. HSQC spectrum of 3.**

**Figure S21. ^1^H-^1^H COSY spectrum of 3.**

**Figure S22. HMBC spectrum of 3.**

**Figure S23. HR-ESI-MS spectrum of 4.**

**Figure S24.** **^1^H NMR spectrum of 4 (600 MHz, CD_3_OD).**

**Figure S25. ^13^C NMR spectrum of 4 (150 MHz, CD_3_OD).**

**Figure S26. HSQC spectrum of 4.**

**Figure S27. ^1^H-^1^H COSY spectrum of 4.**

**Figure S28. HMBC spectrum of 4.**

**Figure S29. HR-ESI-MS spectrum of 5.**

**Figure S30.** **^1^H NMR spectrum of 5 (600 MHz, CD_3_OD).**

**Figure S31. ^13^C NMR spectrum of 5 (150 MHz, CD_3_OD).**

**Figure S32. DEPT 135 spectrum of 5.**

**Figure S33. HSQC spectrum of 5.**

**Figure S34. ^1^H-^1^H COSY spectrum of 5.**

**Figure S35. HMBC spectrum of 5.**

**
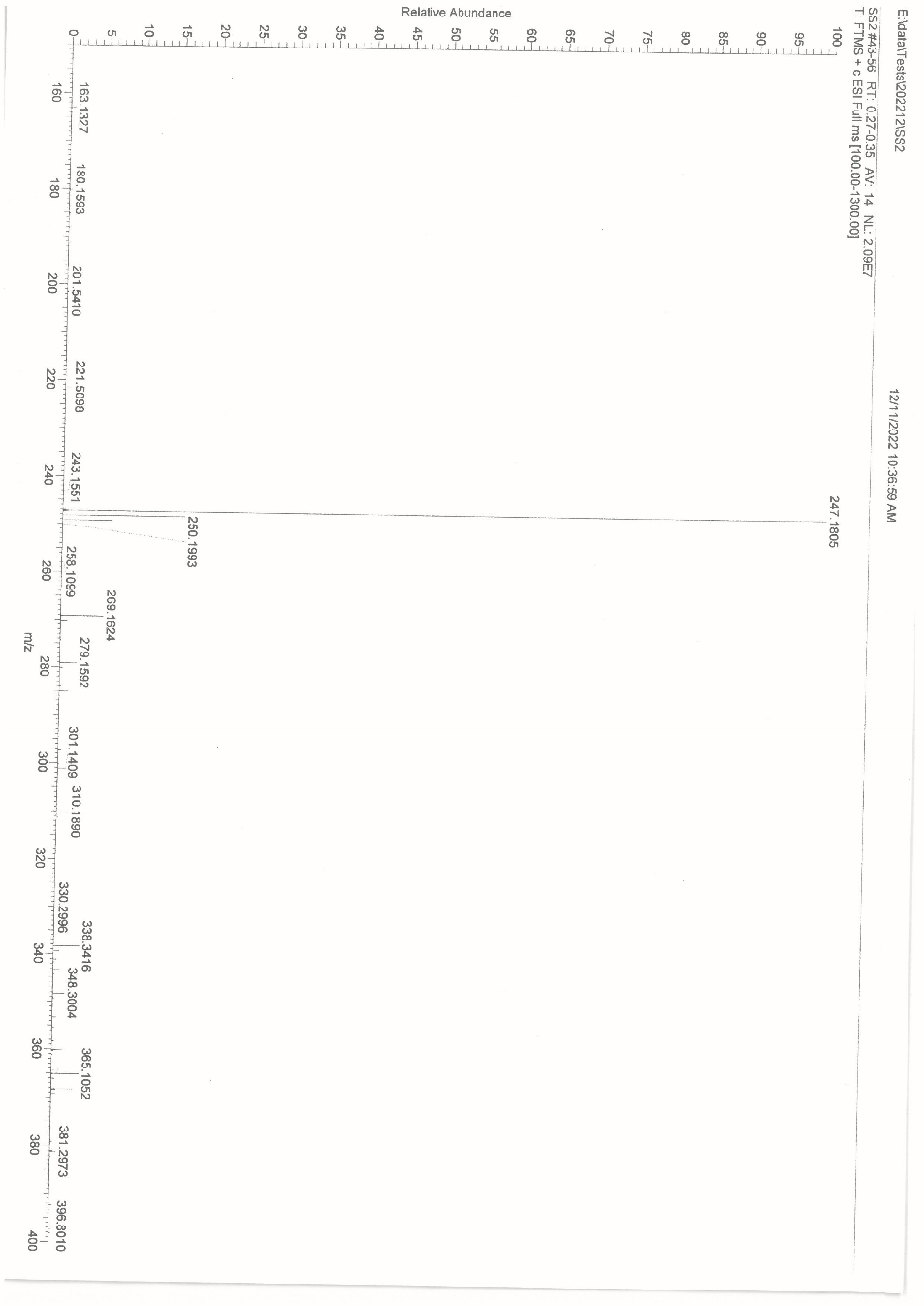
**

**Figure S36. HR-ESI-MS spectrum of 6.**

**Figure S37.** **^1^H NMR spectrum of 6 (600 MHz, CD_3_OD).**

**Figure S38. ^13^C NMR spectrum of 6 (150 MHz, CD_3_OD).**

**Figure S39. DEPT 135 spectrum of 6.**

**Figure S40. HSQC spectrum of 6.**

**Figure S41. ^1^H-^1^H COSY spectrum of 6.**

**Figure S42. HMBC spectrum of 6.**

**Figure S43. NOSEY spectrum of 6.**

**
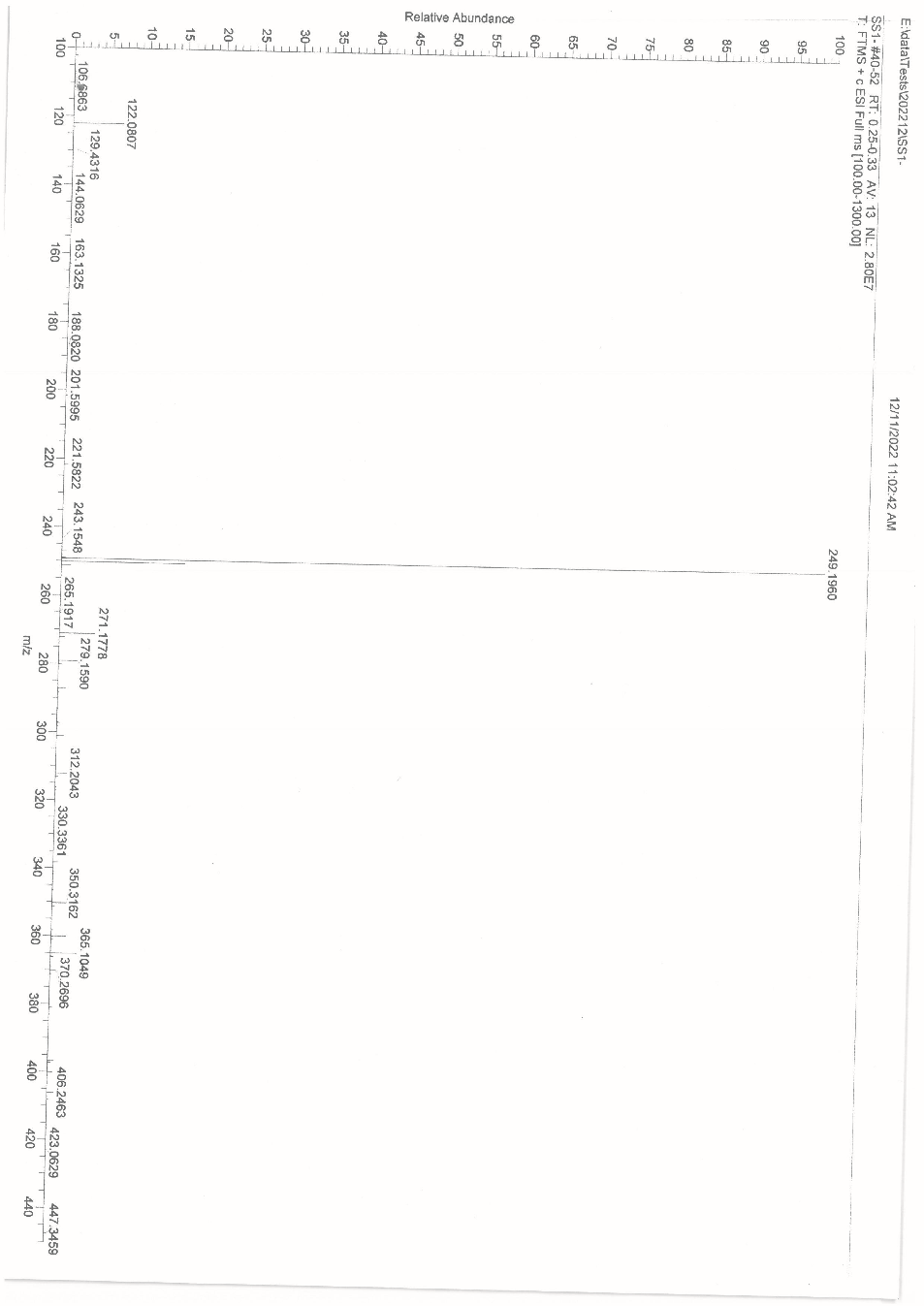
**

**Figure S44. HR-ESI-MS spectrum of 7.**

**Figure S45.** **^1^H NMR spectrum of 7 (600 MHz, CD_3_OD).**

**Figure S46. ^13^C NMR spectrum of 7 (150 MHz, CD_3_OD).**

**Figure S47. DEPT 135 spectrum of 7.**

**Figure S48. HSQC spectrum of 7.**

**Figure S49. ^1^H-^1^H COSY spectrum of 7.**

**Figure S50. HMBC spectrum of 7.**

**Figure S51. NOSEY spectrum of 7.**

**4. Data for serum biochemical indices and blood routines**

**
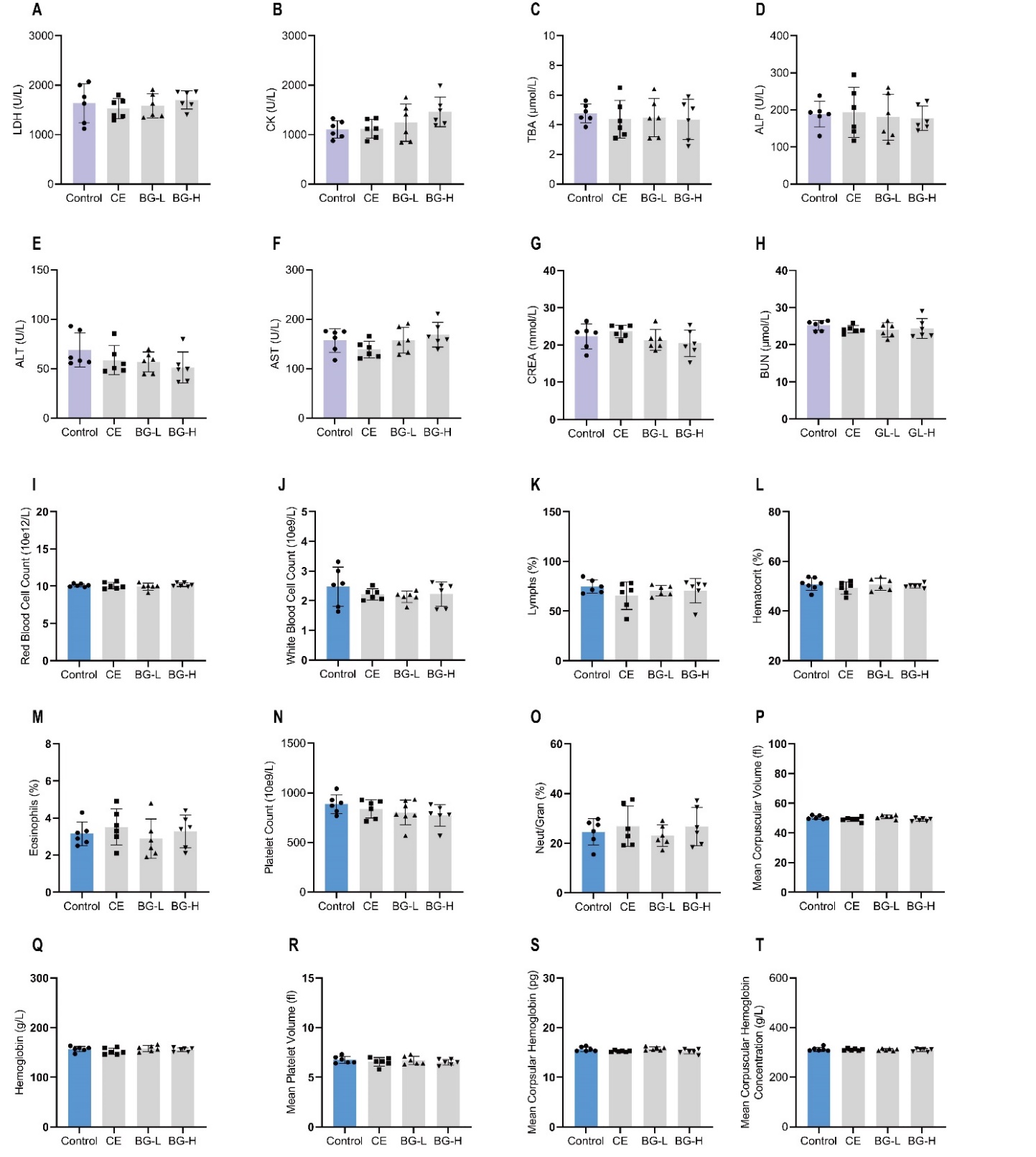
**

**Fig. S52.** Effects of Control, CE, BG-L and BG-H on serum biochemical indices and blood routines in mice in a 28-day subacute toxicity study. (A-B) Myocardial function indicators: creatine kinase (CK) and lactate dehydrogenase (LDH). (C-F) Liver function indicators: total bile acids (TBA), alkaline phosphatase (ALP), alanine transaminase (ALT), and aspartate aminotransferase levels (AST). (I-M) Blood routines parameters reflecting infection and immune function: white blood cell count, red blood cell count, lymphs, eosinophils, neutrophil-to-granulocyte ratio (Neut/Gran). (N-T) Blood routines parameters reflecting anemia, blood viscosity, and platelet function: hemoglobin, hematocrit, mean corpuscular volume, mean platelet volume, mean corpuscular hemoglobin, mean corpuscular hemoglobin concentration and platelet count. Data are illustrated as mean ± SD (n = 6).

**5. Data for RT-PCR**

**Table S1. All primer sequences for RT-PCR.**

| **Gene** | **Primer** | **Sequence of forward and reverse primers (5’ to 3’)** |
| --- | --- | --- |
| *NLRP3* | Forward | 5′-ATTACCCGCCCGAGAAAGG-3' |
|  | Reversed | 5′-TCGCAGCAAAGATCCACACAG-3′ |
| *Caspase-1:* | Forward | 5′-ACAAGGCACGGGACCTATG-3' |
|  | Reversed | 5′-TCCCAGTCAGTCCTGGAAATG-3′ |
| *IL-1β* | Forward | 5′-CAGGATGAGGACATGAGCACC-3′ |
|  | Reversed | 5′-CTCTGCAGACTCAAACTCCAC-3′ |
| *COX-2* | Forward | 5′-CACTACATCCTGACCCACTT-3′ |
|  | Reversed | 5′-ATGCTCCTGCTTGAGTATGT-3′ |
| *β-actin* | Forward | 5′-CGCTCATTGCCGATAGTGAT-3′ |
|  | Reversed | 5′-TGTTTGAGACCTTCAACACC-3′ |

**6. Comparison between known compounds and reported literature**

**Table S2. Known compounds and references**

| **No.** | **Compounds** | **References** |
| --- | --- | --- |
| **8** | kaempferol | [1] |
| **9** | luteolin | [2] |
| **10** | quercetin | [3] |
| **11** | tricin | [4] |
| **12** | astragalin | [5] |
| **13** | isoquercitrin | [6] |
| **14** | rhamnetin-3-*O*-*β*-D-glucopyranoside | [7] |
| **15** | kaempferol-3-*O*-(6′′-*O*-acetyl)-*β*-D-glucopyranoside | [8] |
| **16** | quercetin-3-*O*-(6′′-*O*-acetyl)-*β*-D-glucopyranoside | [9] |
| **17** | kaempferol-3-*O*-(3′′-*O*-acetyl)-*β*-D-glucopyranoside | [10] |
| **18** | tiliroside | [11] |
| **19** | kaempferol-3-*O*-glucorhamnoside | [6] |
| **20** | kaempferol-3-*O*-*α*-L-rhamnopyranosyl-(1→2)-[6-*O*-acetyl]-*β*-D-glucopyranoside | [12] |
| **21** | kaempferol-3-*O*-*α*-L-rhamnopyranosyl-(1→2)-[3-*O*-acetyl]-*β*-D-glucopyranoside | [12] |
| **22** | ajugasterone C | [13] |
| **23** | 20-hydroxyecdysone | [14] |
| **24** | calonysterone | [15] |
| **25** | esculetin | [16] |
| **26** | caffeic acid | [17] |
| **27** | (*E*)-ferulic acid | [18] |
| **28** | (*E*)-*p*-coumaric acid | [19] |
| **29** | syringin | [20] |
| **30** | 4,5-di-*O*-caffeoylquinic acid 1-methyl ether | [21] |
| **31** | geniposide | [22] |
| **32** | phaseic acid | [23] |
| **33** | uridine | [24] |
| **34** | 1-(*β*-D-ribofuranosyl)-1*H*-1,2,4-triazone | [25] |
| **35** | 3-hydroxypyridine | [26] |
| **36** | methyl-5-hydroxypyridine-2-carboxlate | [27] |
| **37** | isohematinic acid | [28] |
| **38** | uracil | [29] |
| **39** | protocatechuic acid | [30] |
| **40** | *p*-hydroxybenzoic acid | [31] |
| **41** | vanillic acid | [32] |
| **42** | *p*-hydroxyphenethyl alcohol | [33] |
| **43** | gallic acid | [32] |
| **44** | 3,4-dihydroxybenzyl alcohol | [34] |
| **45** | 1*H*-indole-3-carboxaldehyde | [35] |
| **46** | (+)-syringaresinol | [36] |
| **47** | lirioresionol | [37] |
| **48** | (+)-medioresinol | [38] |
| **49** | (+)-pinoresinol | [39] |
| **50** | 5-methoxy-(+)-isolariciresinol | [40] |
| **51** | (+)-isolariciresinol | [40] |
| **52** | (+)-lyoniresinol | [41] |
| **53** | (7*S*, 8*R*)-dihydrodehydro-  diconiferyl alcohol | [42] |
| **54** | 5-methoxydehydro-  coniferyl alcohol | [43] |
| **55** | isoscopoletin | [44] |
| **56** | scopoletin | [45] |
| **57** | isofraxidin | [46] |
| **58** | (+)-dehydrovomifoliol | [47] |
| **59** | (−)-loliolide | [48] |
| **60** | (+)-isololiolide | [49] |
| **61** | *p*-hydroxyacetophenone | [50] |
| **62** | dihydroconiferylalcohol | [16] |
| **63** | acetovanillone | [51] |

**7. Data for network pharmacology analysis**

**Table S3. Active** **components screened using Swiss target ADME.**

| **Rank** | **Ingredients** |
| --- | --- |
| 1 | Quercetin (**10**) |
| 2 | Luteolin (**9**) |
| 3 | Kaempferol (**8**) |
| 4 | Apigenin |
| 5 | Chrysoeriol |
| 6 | Tricin (**11**) |
| 7 | Sophocarpine |
| 8 | Thesiumine B (**7**) |
| 9 | Thesiumine A (**6**) |
| 10 | Uridine (**33**) |

**Table S4. Common genes of active components and drug targets.**

| **Common genes** | | | | | | | | |
| --- | --- | --- | --- | --- | --- | --- | --- | --- |
| AR | CDK4 | ABCC1 | PGR | IL10 | COL1A1 | MPO | ABCG2 | IL4 |
| CHRM3 | MCL1 | NOS2 | MAPK8 | MDM2 | COL3A1 | MYC | RASSF1 | CDKN2A |
| PARP1 | AKT1 | PTGS1 | SELE | MET | CRP | NFE2L2 | CHEK2 | IRF1 |
| BCHE | XIAP | PTGS2 | SLPI | MMP2 | NQO1 | NOS3 | NCF1 | SPP1 |
| CYP2D6 | HSP90AA1 | DPP4 | TNF | MMP9 | E2F1 | ODC1 | MIR21 | CAT |
| MAPK14 | MAPK1 | CCNA2 | VCAM1 | NFKBIA | EGF | SERPINE1 | CTNNB1 | ESR1 |
| IL1A | RAF1 | ESR2 | NR1I2 | PCNA | ERBB3 | PLAT | PLA2G2A | MMP1 |
| CXCL10 | BCL2 | AHR | AHSA1 | MAPK3 | F3 | PLAU | STAT3 | IL6 |
| IRAK1 | SRC | ALOX5 | CDK1 | RB1 | FOS | PON1 | CYP1A1 | CHUK |
| NFKB1 | RIPK1 | BAX | BIRC5 | TOP1 | GJA1 | POR | IKBKB | MMP3 |
| RELA | BCL2L1 | CASP3 | CCND1 | TOP2A | CXCL2 | PPARA | ERBB2 | THBD |
| PPARG | CD274 | CYP1B1 | CASP7 | VEGFA | HIF1A | PRKCA | CAV1 |  |
| SIRT1 | IFNG | CYP3A4 | CASP9 | PTGES | HSPA5 | PTEN | CXCL8 |  |
| SMAD2 | IL2 | GSTM1 | CCNB1 | ADRB2 | HSPB1 | PTGER3 | SOD3 |  |
| SMAD3 | STAT1 | GSTP1 | CD40LG | AKR1B1 | IGF2 | RASA1 | TP53 |  |
| TRPV4 | LEP | HMOX1 | CDKN1A | BMP2 | IGFBP3 | CCL2 | CYP1A2 |  |
| TGFB1 | CDK2 | ICAM1 | EGFR | CASP8 | IL1B | SOD1 | JUN |  |

**8. References**

1. Guzmán-Gutiérrez SL, Nieto-Camacho A, Castillo-Arellano JI et al. Mexican propolis: a source of antioxidants and anti-inflammatory compounds, and isolation of a novel chalcone and ε-caprolactone derivative. Molecules. 2018;23(2):16.

2. Ma XQ, Zheng CJ, Zhang Y et al. Antiosteoporotic flavonoids from *Podocarpium podocarpum*. Phytochem Lett. 2013;6(1):118-22.

3. Devkota HP, Tsushiro K, Watanabe T. Bioactive phenolic compounds from the flowers of *Farfugium japonicum* (L.) Kitam. var. *giganteum* (Siebold et Zucc.) Kitam. (Asteraceae). Nat Prod Res. 2022;36(15):4036-39.

4. Duc Hung N, Zhao BT, Duc Dat L et al. Phenolic constituents and their anti-inflammatory activity from *Echinochloa utilis* Grains. Nat Prod Sci. 2016;22(2):140-45.

5. Kishore PH, Reddy MVB, Gunasekar D et al. A new coumestan from *Tephrosia calophylla*. Chem Pharm Bull (Tokyo). 2003;51(2):194-96.

6. Kazuma K, Noda N, Suzuki M. Malonylated flavonol glycosides from the petals of *Clitoria ternatea*. Phytochemistry. 2003;62(2):229-37.

7. He ZN, Lian WW, Liu JW et al. Isolation, structural characterization and neuraminidase inhibitory activities of polyphenolic constituents from *Flos caryophylli*. Phytochem Lett. 2017;19:160-67.

8. Tai ZG, Zhang FM, Cai L et al. Flavonol glycosides of *Pseudodrynaria coronans* and their antioxidant activity. Chem Nat Compd. 2012;48(2):221-24.

9. Merfort I, Wendisch D. Flavonoid Glycosides from *Arnica montana* and *Arnica chamissionis*. Planta Med. 1987(5):434-37.

10. Zaghloul AM. Investigation of the flavonoid content of *verbena peruviana*. Mansoura J Pharm Sci. 1995;11(1):43-54.

11. Lee J, Kim NH, Nam JW et al. Scopoletin from the flower buds of *Magnolia fargesii* inhibits protein glycation, aldose reductase, and cataractogenesis *ex vivo*. Arch Pharmacal Res. 2010;33(9):1317-23.

12. Wu H, Dushenkov S, Ho CT et al. Novel acetylated flavonoid glycosides from the leaves of *Allium ursinum*. Food Chem. 2009;115(2):592-95.

13. Aliouche L, Larguet H, Amrani A et al. Isolation, antioxidant and antimicrobial activities of ecdysteroids from *Serratula cichoracea*. Curr Bioact Compd. 2018;14(1):60-66.

14. Zughdani M, Yusufoglu HS, Ekiz G et al. Ecdysteroids from the underground parts of *Rhaponticum acaule* (L.) DC. Phytochemistry. 2020;180:11.

15. Csabi J, Hsieh TJ, Hasanpour F et al. Oxidized metabolites of 20-hydroxyecdysone and their activity on skeletal muscle cells: preparation of a pair of desmotropes with opposite bioactivities. J Nat Prod. 2015;78(10):2339-45.

16. Huang YH, Zeng WM, Li GY et al. Characterization of a new sesquiterpene and antifungal activities of chemical constituents from *Dryopteris fragrans* (L.) Schott. Molecules. 2014;19(1):507-13.

17. Zhou LG, Li D, Wang JG et al. Antibacterial phenolic compounds from the spines of *Gleditsia sinensis* Lam. Nat Prod Res. 2007;21(4):283-91.

18. Seki T, Morimura S, Tabata S et al. Antioxidant activity of vinegar produced from distilled residues of the Japanese liquor shochu. J Agric Food Chem. 2008;56(10):3785-90.

19. Goetz G, Fkyerat A, Metais N et al. Resistance factors to grey mould in grape berries: identification of some phenolics inhibitors of *Botrytis cinerea* stilbene oxidase. Phytochemistry. 1999;52(5):759-67.

20. Wang L, Yang J, Chi YQ et al. A new myrsinol-type diterpene polyester from *Euphorbia dracunculoides* Lam. Nat Prod Res. 2015;29(15):1406-13.

21. Ela MAA, El-Lakany AM, Abdel-Kader MS et al. New quinic acid derivatives from hepatoprotective *Inula crithmoides* root extract. Helv Chim Acta. 2012;95(1):61-66.

22. Ono M, Ueno M, Masuoka C et al. Iridoid glucosides from the fruit of *Genipa americana*. Chem Pharm Bull (Tokyo). 2005;53(10):1342-44.

23. Hirai N, Kondo S, Ohigashi H. Deuterium-labeled phaseic acid and dihydrophaseic acids for internal standards. Biosci, Biotechnol, Biochem. 2003;67(11):2408-15.

24. Sang SM, Kikuzaki H, Lapsley K et al. Sphingolipid and other constituents from almond nuts (*Prunus amygdalus* Batsch). J Agric Food Chem. 2002;50(16):4709-12.

25. Huang RM, Zhou XF, Peng Y et al. Nucleosides from the marine sponge *Callyspongia* sp. Chem Nat Compd. 2011;46(6):1010-11.

26. Chou CH, Chu LT, Chen IY et al. Pyrolytic study of cyclic 2-azidoketones. Heterocycles. 2008;75(3):577-82.

27. Deady L, Shanks R, Campbell A et al. The synthesis of some substituted methyl pyridinecarboxylates. II. Methyl 4-substituted picolinates, methyl 5-substituted picolinates, and methyl 5-substituted nicotinates Aust J Chem. 1971;24(2):385-92.

28. Itoh Y, Takeuchi M, Shimizu K et al. New antibiotic, isohematinic acid. II. Physico-chemical properties, structural elucidation and biological activities. J Antibiot. 1983;36(5):497-501.

29. Rathnayake GRN, Kumar NS, Jayasinghe L et al. Chemical investigation of metabolites produced by an endophytic fungi *Phialemonium curvatum* from the leaves of *Passiflora edulis*. Nat Prod Res. 2018;32(20):2483-86.

30. Ourhzif EM, Ricelli A, Stagni V et al. Antifungal and cytotoxic activity of diterpenes and bisnorsesquiterpenoides from the latex of *Euphorbia resinifera* Berg. Molecules. 2022;27(16):15.

31. Yuan X, Wen H, Cui Y et al. Phenolics from *Lagotis brevituba* Maxim. Nat Prod Res. 2017;31(3):362-66.

32. Zhang YJ, DeWitt DL, Murugesan S et al. Novel lipid-peroxidation- and cyclooxygenase-inhibitory tannins from *Picrorhiza kurroa* seeds. Chem Biodivers. 2004;1(3):426-41.

33. Ida Y, Satoh Y, Ohtsuka M et al. Phenolic constituents of *Phellodendron amurense* bark. Phytochemistry. 1994;35(1):209-15.

34. Zhang ZZ, Xiao BH, Chen Q et al. Synthesis and biological evaluation of caffeic acid 3,4-dihydroxyphenethyl ester. J Nat Prod. 2010;73(2):252-54.

35. Ren L, Wang YZ, Zhang W et al. Triculata A, a novel compound from *Tricyrtis maculata* (D. Don) J. F. Macbr. with biological properties. Nat Prod Res. 2021;35(21):3729-37.

36. Park HB, Lee KH, Kim KH et al. Lignans from the roots of *Berberis amurensis*. Nat Prod Sci. 2009;15(1):17-21.

37. Briggs LH, Cambie RC, Couch RAF. Lirioresinol-C dimethyl ether, a diaxially substituted 3,7-dioxabicyclo [3,3,0]octane lignan from *Macropiper excelsum* (Forest. f.) Miq. J Chem Soc C. 1968(24):3042-&.

38. Abe F, Yamauchi T. 9*α*-Hydroxypinoresinol, 9*α*-hydroxymedioresinol and related lignans from *Allamanda neriifolia*. Phytochemistry. 1988;27(2):575-77.

39. Moon SS, Rahman AA, Kim JY et al. Hanultarin, a cytotoxic lignan as an inhibitor of actin cytoskeleton polymerization from the seeds of *Trichosanthes kirilowii*. Biorg Med Chem. 2008;16(15):7264-69.

40. Jutiviboonsuk A, Zhang HJ, Tan GT et al. Bioactive constituents from roots of *Bursera tonkinensis*. Phytochemistry. 2005;66(23):2745-51.

41. Choi SE, Yoon JH, Choi HK et al. Phenolic compounds from the root of *Phragmites communis*. Chem Nat Compd. 2009;45(6):893-95.

42. Kuang HX, Xia YG, Yang BY et al. Lignan constituents from *Chloranthus japonicus* Sieb. Arch Pharmacal Res. 2009;32(3):329-34.

43. Li LY, Seeram NP. Maple syrup phytochemicals include lignans, coumarins, a stilbene, and other previously unreported antioxidant phenolic compounds. J Agric Food Chem. 2010;58(22):11673-79.

44. Zhao DD, Zhao QS, Liu L et al. Compounds from *Dryopteris Fragrans* (L.) Schott with cytotoxic activity. Molecules. 2014;19(3):3345-55.

45. Chaves OS, Teles YCF, Monteiro MMD et al. Alkaloids and phenolic compounds from *Sida rhombifolia* l. (malvaceae) and vasorelaxant activity of two indoquinoline alkaloids. Molecules. 2017;22(1):9.

46. Panichayupakaranant P, Noguchi H, Deeknamkul W et al. Naphthoquinones and coumarins from *Impatiens balsamina* root cultures. Phytochemistry. 1995;40(4):1141-43.

47. Park JH, Lee DG, Yeon SW et al. Isolation of megastigmane sesquiterpenes from the silkworm (*Bombyx mori* l.) droppings and their promotion activity on HO-1 and SIRT1. Arch Pharmacal Res. 2011;34(4):533-42.

48. De Marino S, Borbone N, Gala F et al. New constituents of sweet *Capsicum annuum* L. fruits and evaluation of their biological activity. J Agric Food Chem. 2006;54(20):7508-16.

49. Xu W, Wang JH, Ju BZ et al. Seven compounds from *Portulaca oleracea* L. and their anticholinesterase activities. Nat Prod Res. 2022;36(10):2547-53.

50. Xiu F, Li XT, Zhang WJ et al. A new alkaloid from *Portulaca oleracea* L. and its antiacetylcholinesterase activity. Nat Prod Res. 2019;33(18):2583-90.

51. Luo JR, Jiang HE, Zhao YX et al. Components of the heartwood of *Populus euphratica* from an ancient tomb. Chem Nat Compd. 2008;44(1):6-9.
